# Unveiling the Transgalactosylation Switch of a GH42 β‑Galactosidase from the Infant Isolate *Bifidobacterium breve* DSM20213

**DOI:** 10.1021/acscatal.5c08164

**Published:** 2026-01-23

**Authors:** Konlarat Phirom-on, Khanh-Trang Vu-Le, Leander Sützl, Benedikt Lehner, David Whelan, Lucile Guerent, Irene Pasini, Marc Schuh, Anita de Ruiter, Markus Blaukopf, Dietmar Haltrich, Chris Oostenbrink, Thu-Ha Nguyen

**Affiliations:** † Food Biotechnology Laboratory, Institute of Food Technology, Department of Biotechnology and Food Science, 27270 BOKU University, Muthgasse 18, A-1190 Vienna, Austria; ‡ Doctoral Programme BioToP-Biomolecular Technology of Proteins, BOKU University, Muthgasse 18, A-1190 Vienna, Austria; § Institute of Organic Chemistry, Department of Natural Sciences and Sustainable Resources, BOKU University, Muthgasse 18, A-1190 Vienna, Austria; ∥ Institute for Molecular Modeling and Simulation, Department of Natural Sciences and Sustainable Resources, BOKU University, Muthgasse 18, A-1190 Vienna, Austria; ⊥ Faculty of Biology and Environmental Science, The University of Danang - University of Science and Education, 550000 Danang, Vietnam

**Keywords:** transgalactosylation, galacto-oligosaccharides (GOS), β-galactosidase, glycoside hydrolase (GH) family 42, protein engineering, water tunnel

## Abstract

β-Galactosidases catalyze the transgalactosylation of lactose to produce prebiotic galacto-oligosaccharides (GOS), key fortificants in infant formulas. A β-galactosidase from the infant isolate *Bifidobacterium breve* DSM20213 (*Bbre*βgal-III), which belongs to the glycoside hydrolase (GH) family 42, exhibits limited transgalactosylation activity, resulting in a low yield of GOS with the predominant formation of β-(1→6)-linked GOS. Structural analysis revealed a hydrophobic-rich active site and the presence of a water tunnel connecting the deeply buried active site to the exterior environment. The highly conserved hydrophilic Arg121 residue, which is adjacent to the catalytic acid/base residue Glu160, was found to play a crucial role in the hydrolytic activity of *Bbre*βgal-III. The guanidino group of the Arg121 side chain forms a network of hydrogen bonds involving the catalytic Glu160 and a water molecule in the water tunnel. This influences the coordination environment of the active site, resulting in a preference for hydrolysis over transgalactosylation in *Bbre*βgal-III. Site-saturation mutagenesis at Arg121 revealed that all variants enhanced the transgalactosylation activity. Among these, *Bbre*βgal-III-R121C exhibited the highest GOS yield, reaching 34% mass of total sugars in the transgalactosylation reaction compared to 17% by the wild-type enzyme. *Bbre*βgal-III-R121C not only enhanced transgalactosylation but also displayed distinct changes in the main GOS components synthesized, including a shift from 6′-galactosyllactose, which is formed predominantly by wild-type *Bbre*βgal-III, to 3′-galactosyllactose and a notable increase in the formation of β-(1→2)-linked GOS. These results suggest that Arg121 acts as a key switch for transgalactosylation/hydrolysis activity in GH42 β-galactosidases. Furthermore, water tunnel engineering, i.e., modification of the active-site access pathway, using alanine scanning also increased the transgalactosylation activity. Disrupting the movement of water molecules within the tunnel resulted in higher transgalactosylation activity. Understanding the catalytic importance of amino acids involved in transgalactosylation and rational mutagenesis of active-site residues provided further insights into the structure−function relationships of β-galactosidases within the GH42 family.

## Graphical abstract

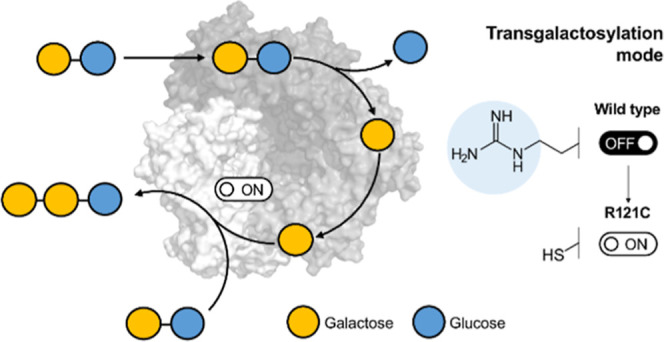

## Introduction

β-Galactosidases catalyze both the hydrolysis and transgalactosylation of lactose, of which the latter is of biotechnological interest for the biosynthesis of galacto-oligosaccharides (GOS), the dominant functional food ingredients fulfilling the criteria of “prebiotics”. The transgalactosylation reaction involves the transfer of the galactosyl moiety of lactose (as galactosyl donor) to suitable sugar acceptors, which can be glucose, galactose, lactose itself, or any sugar species present in the reaction mixture, depending on the availability of these sugars during lactose conversion. The transgalactosylation reaction catalyzed by β-galactosidases is more preferable for the biosynthesis of GOS^1–3^ than the reaction using glycosyltransferases due to limited supply, high price, and necessity of specific sugar nucleotides as substrate for the latter enzymes.

The structural building blocks of GOS mixtures depend on the specificity in linkage formation of the β-galactosidases used in the transgalactosylation reaction. Among microbial β-galactosidases, glycoside hydrolase (GH) family 2 β-galactosidases are the most studied and well-characterized enzymes for the transgalactosylation reaction to biosynthesize GOS.^2,4–6^ The β-(1→4)-linked GOS are predominant in commercial V-GOS produced using a GH2 β-galactosidase from *Bacillus circulans*,^7,8^ whereas GH2 β-galactosidases from lactic acid bacteria and *Bifidobacterium breve* show preference to form β-(1→6)- and β-(1→3)-linked GOS.^2^ Owing to their different substrate specificities, β-galactosidases of GH2 and GH42 are often found in the same organism.^9–11^ However, the catalytic efficiency of GH42 β-galactosidases in transgalactosylation is still debated. Some studies of GH42 β-galactosidases showed no transgalactosylation,^12–15^ while others reported that transgalactosylated products could be obtained.^1,16^

Members of the genus *Bifidobacterium* are one of the most common organisms found in the human gastro-intestinal tract.^17,18^ Among bifidobacteria, mainly, *B. longum* subsp. *infantis*, *B. longum* subsp. *longum*, *B. breve,* and *B. bifidum* dominate in the gut of breast-fed infants.^19^ Human milk can serve as the sole source of nourishment for breast-fed infants, and human milk oligosaccharides (HMO) are prominent among the functional components of human milk. Infant-associated *Bifidobacterium* species appear to have developed different strategies for degrading HMO as they are equipped with genetic and enzymatic sets dedicated to the utilization of HMO.^20^

While *B. longum* subsp. *infantis* is the most studied species in terms of genetic adaptations to utilize HMO,^21–25^ less attention has been drawn to *B. breve* regarding HMO utilization, although it is also a dominant species in the infant gut. The enzymes found in different strains of *B. breve*, which are reported to take part in the degradation of HMO, include a GH33 α-sialidase,^22^ a GH29 α-fucosidase,^21,22^ a GH42 β-galactosidase, and two GH2 β-galactosidases,^26^ of which the β-galactosidases display the abilities to hydrolyze type I and type II backbone structures of HMO. We are interested in studying these β-galactosidases from *B. breve* DSM20213, an isolate from the infant gut, to better understand their mechanisms in the degradation of HMO as well as to investigate them for the biosynthesis of GOS. We previously reported on two GH2 β-galactosidases (*Bbre*βgal-I, BBBR_0011; *Bbre*βgal-II, BBBR_1549) from *B. breve* DSM20213 (GenBank genome sequence AP012324.1) regarding their biochemical properties for the formation of GOS in transgalactosylation mode,^5^ as well as their propensity to transfer the galactosyl moiety onto various sugars.^3^ *Bbre*βgal-I and *Bbre*βgal-II were found to be very well suited for the production of GOS with total obtained yields of 33% and 44% mass of total sugars, respectively.^5^ In contrast, the GH42 β-galactosidase (*Bbre*βgal-III, BBBR_0453) from *B. breve* DSM20213 displays considerably lower transgalactosylation activity, resulting in a relatively low GOS yield of only 17%.^27^

Several studies attempted to improve transglycosylation through protein engineering by modifying the active site and water dynamics within the protein structure to favor the transglycosylation reaction over hydrolysis.^28,29^ A GH42 β-galactosidase from *Alicyclobacillus acidocaldarius* was successfully engineered into a glycosynthase-like variant, which shows efficient transglycosylation activity assisted by sodium azide and sodium formate.^30^ In this study, we provide a comprehensive investigation of GH42 *Bbre*βgal-III from *B. breve* DSM20213 at a molecular level. Various strategies to identify factors hindering transgalactosylation and to activate the transgalactosylation switch of *Bbre*βgal-III are described. Structural insights into the active site of *Bbre*βgal-III enable the identification of residues potentially important for transgalactosylation and, thus, for engineering of the enzyme. The shift in the primary synthesized GOS products, from β-(1→6)- to β-(1→3)-linked GOS, and the increase in the formation of β-(1→2)-linked GOS render the engineered variant *Bbre*βgal-III-R121C an interesting biocatalyst, which favors the formation of specific GOS structures with increased yields.

## Results

### Structural Analysis of *Bbre*βgal-III

The predicted protein structure of *Bbre*βgal-III (Figure 1A top-left) was obtained from AlphaFold3, with a pLDDT >90, an iPTM of 0.95, and a pTM of 0.96, indicating a highly reliable structure.^31^ It was subsequently overlaid with a crystal structure of a GH42 β-galactosidase from *B. longum* subsp. *infantis* ATCC 15697 (*Bi*Bga42A; PDB: 8IBT), which shows a 95% sequence identity with *Bbre*βgal-III, resulting in a final RMSD of 0.45 Å over 654 Cα atoms. This structure also contained the tetrasaccharide β-d-Gal-(1→3)-β-d-GlcNAc-(1→3)-β-d-Gal-(1→4)-d-Glc (lacto-*N*-tetraose, LNT) as a ligand. Hence, it was possible to identify the active site of *Bbre*βgal-III. The ligand is bound within domain 1, which shows a TIM-barrel fold. *Bbre*βgal-III forms a homotrimeric structure in its native state organized in three identical subunits (Figure 1A top-right). The interactions between the surface of the subunits create three cavities, of which each serves as an active site, resulting in three identical active sites in *Bbre*βgal-III. Structural prediction of *Bbre*βgal-III also suggests that the galactose moiety in LNT at the nonreducing end is in the −1 subsite, which is the binding site for the sugar donor in the transgalactosylation reaction (Figure 1A bottom-left). *N*-Acetylglucosamine, galactose, and glucose occupy the +1, +2, and +3 subsites, respectively, which together form the sugar acceptor subsite. Further analysis of the structural model revealed a long tunnel connecting the active site to the back of the enzyme, with a diameter smaller than 3 Å, indicating that only water molecules are able to pass through.

**Figure 1:**
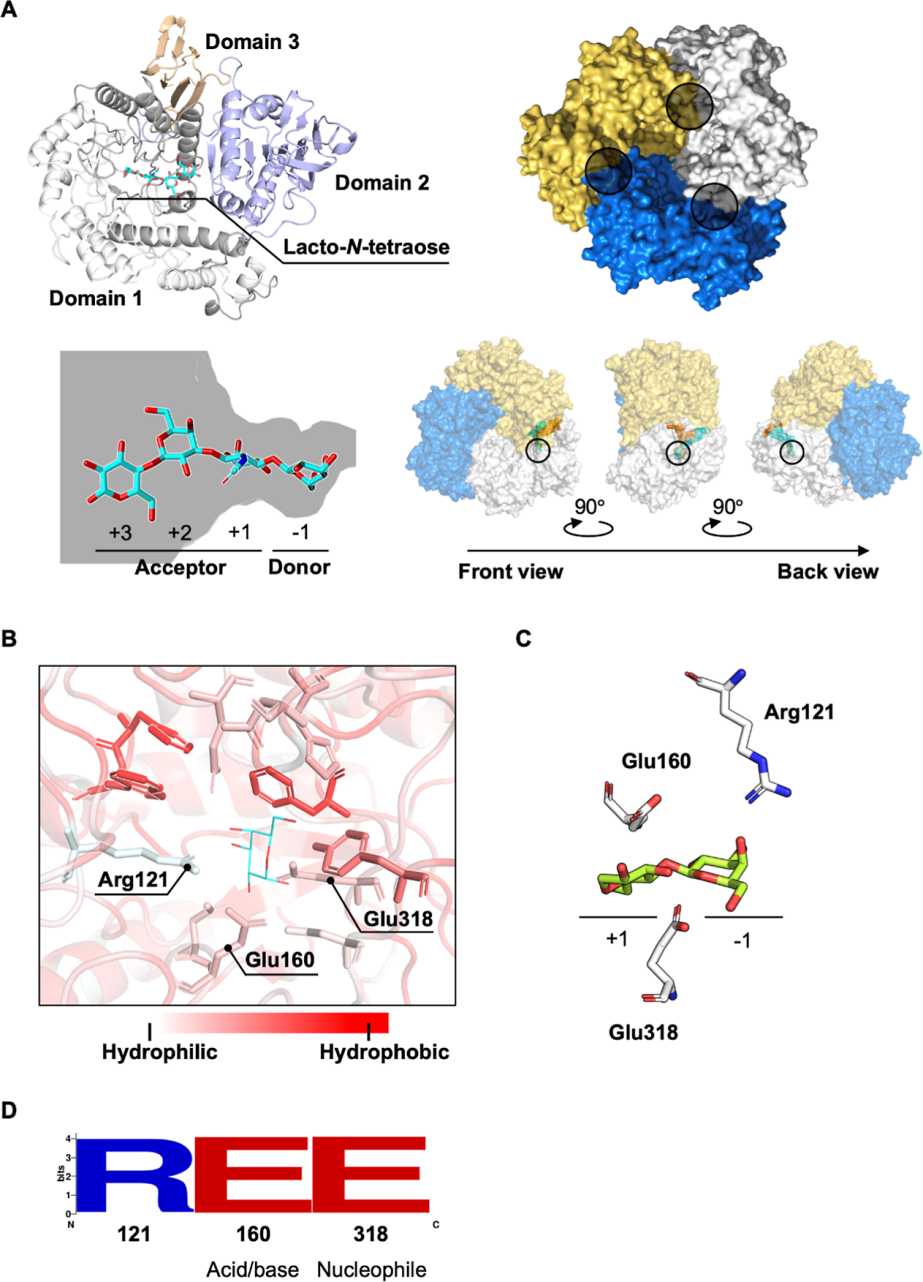
Predicted structure of the GH42 *Bbre*βgal-III from *B. breve*. (A) predicted structure of *Bbre*βgal-III from *B. breve* DSM 20213 as predicted by AlphaFold3. Top-left: monomeric *Bbre*βgal-III subunit showing three domains (catalytic domain 1, gray; trimerization domain 2, purple; domain 3, orange) and β-d-Gal-(1→3)-β-d-GlcNAc-(1→3)-β-d-Gal-(1→4)-d-Glc (lacto-*N*-tetraose, cyan) as a ligand; top-right: quaternary structure of *Bbre*βgal-III indicating three identical subunits; the individual active sites are marked (within the circles); bottom-left: schematic representation of the active site with lacto-*N*-tetraose as sugar donor and acceptor binding subsites; bottom-right: the trimeric structure of wild-type *Bbre*βgal-III at different angles showing a long buried water tunnel connecting the active site to the exterior environment. The black circle indicates the location of the active site. (B) Hydrophobicity of the amino acid residues surrounding the sugar donor subsite in the active site of *Bbre*βgal-III with docked α-galactose. (C) The sugar subsites in the active site of GH42 *Bbre*βgal-III with docked β-d-Gal-(1→4)-d-Glc (lactose, green). The d-galactose moiety is positioned in the sugar donor subsite (−1 subsite), while d-glucose occupies the sugar acceptor subsite (+1 subsite). Arg121 is located within the −1 subsite and is proposed to participate in the transgalactosylation/hydrolysis mechanism. Glu160 and Glu380 serve as the catalytic acid/base and nucleophile residues, respectively. Lactose docking was performed using Molecular Operating Environment (MOE). (D) Sequence logo analysis of GH42 β-galactosidases in the Uniprot database highlighting high conservation levels of Arg121, acid/base (Glu160), and nucleophile (Glu318) residues.

Based on these findings, tunnels were also analyzed in molecular dynamics simulation of the protein. The simulations revealed two tunnels connecting the active site to the back of the enzyme, which were detected in all of the three cavities (Figure 1A bottom-right). The characterization of the water tunnels is discussed later in the section on the structural analysis of the water tunnels in *Bbre*βgal-III.

Initially, we focused on engineering the active site of *Bbre*βgal-III. The superimposed structure of the enzyme’s active site revealed a hydrophobic pocket surrounding the galactose molecule at the −1 subsite (Figure 1B). Interestingly, in addition to the catalytic residues, there is only one single hydrophilic residue, Arg121, located in this pocket. When lactose was docked in the active site of *Bbre*βGal-III, the d-galactose moiety is positioned in the sugar donor subsite (−1 subsite), while d-glucose occupies the sugar acceptor subsite (+1 subsite). Arg121 is located within the −1 subsite (Figure 1C). Sequence alignment of 301 characterized GH42 β-galactosidases available in the UniProt database revealed a high conservation of Arg121 (Figure 1D). The overlaid structure of the active site of *Bbre*βgal-III with the active sites of the GH42 *Bi*Bga42A from *B. longum* subsp. *infantis* ATCC 15697 (PDB: 8IBT) and GH42 BbgII from *B. bifidum* S17 (PDB: 4UCF) (Figure S1) also indicates the conservation of Arg121 as well as the two catalytic glutamic acid residues, Glu160 and Glu318. The high level of conservation of Arg121 was comparable with the conservation levels of the catalytic residues Glu160 (acid/base) and Glu318 (nucleophile). This suggests a critical role of Arg121 in GH42 β-galactosidases, making it an interesting active-site residue to investigate.

### Site-Saturation Mutagenesis of Arg121

Site-saturation mutagenesis of Arg121 in *Bbre*βgal-III was conducted, and the resulting variants were expressed in *Escherichia coli* and purified using a prepacked HisTrap HP Ni-immobilized metal ion affinity chromatography (IMAC) 5 mL column (Cytiva, MA, USA); see in Figure S2. During lactose conversion, glucose is released during the first reaction step (glycosylation), and the rate of this release from the enzyme-substrate complex is termed the global activity (Figure 2A). Galactose is then released in the second reaction step (deglycosylation) if water acts as a nucleophile and reacts with the galactosyl-enzyme intermediate in a process called hydrolysis. In transgalactosylation, a sugar species acts as a nucleophile instead of a water molecule and attacks the anomeric carbon of the galactosyl-enzyme intermediate, resulting in the formation of the galactosyl adduct. The partitioning of the galactosyl-enzyme intermediate (E-Gal in Figure 2A) between hydrolysis and transgalactosylation can be studied under initial velocity conditions.^3^

**Figure 2:**
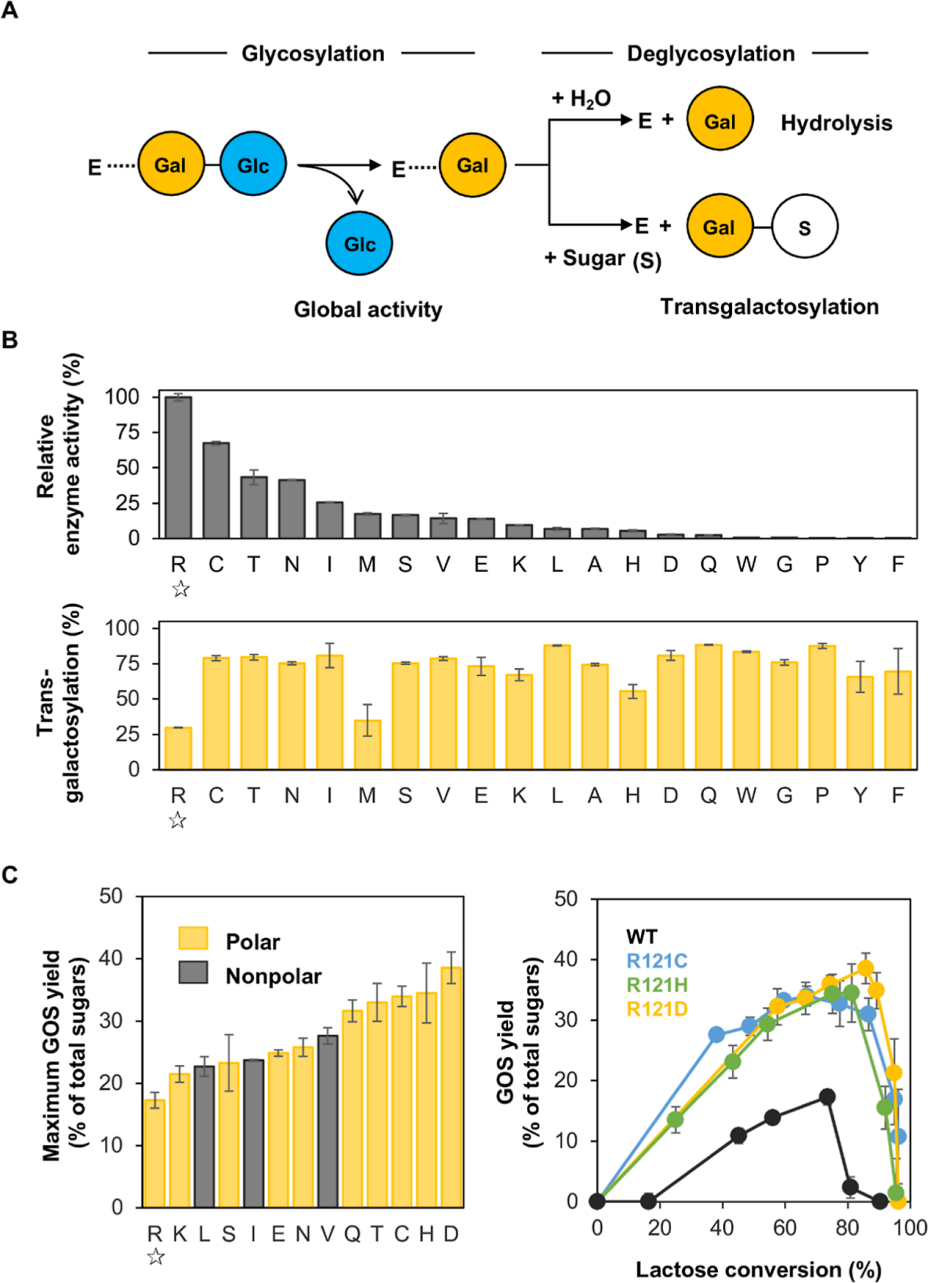
Transgalactosylation and yields of GOS formed by *Bbre*βgal-III Arg121 variants. (A) Enzymatic reaction scheme of wild-type *Bbre*βgal-III with lactose as substrate. Release of glucose (Glc) and the formation of a covalent galactosyl-enzyme intermediate (E-Gal) in the first reaction step (or glycosylation step); hydrolysis of the galactosyl-enzyme intermediate (E-Gal) leading to the release of galactose (Gal) or the transfer of Gal to a sugar acceptor (S) other than water (hydrolysis *vs* transglycosylation) in the second reaction step (deglycosylation step). (B) Relative enzyme activities and transgalactosylation activities of R121 variants compared to wild-type *Bbre*βgal-III (marked as star open); top: specific enzyme activities (U/mg) refer to global activities in the glycosylation step. Reactions were performed using 600 mM lactose in 50 mM NaPB (pH 6.5) at 30 °C for 10 min, and the release of d-glucose was measured (see Materials and Methods). The activities are shown as relative enzyme activities to the wild-type. Bottom: transgalactosylation activities refer to the direction of E-Gal toward transgalactosylation. A reaction mixture containing ∼2.5 U/mL of enzyme was incubated with 600 mM lactose in 50 mM NaPB (pH 6.5) at 30 °C for 10 min. The concentrations of d-glucose and d-galactose in the reaction mixture were measured, and the transgalactosylation activity was calculated according to eq 2 (see Materials and Methods). (C) Left: maximum GOS yields of selected R121 variants; the data were statistically analyzed using one-way ANOVA (*p* value is 0.99). Right: GOS yields obtained during lactose conversions by wild-type *Bbre*βgal-III (WT) and three variants R121C, R121H, and R121D. Reaction mixtures for lactose conversion containing 2.5 U/mL of enzyme and 600 mM lactose were incubated at 37 °C for 24 h. d-Glucose, d-galactose, and d-lactose were measured, and the GOS formed was calculated using eq 3 (see Materials and Methods).

The enzyme activity, here termed global activity (eq 1 in Materials and Methods), was compared in terms of specific activity of the Arg121 variants relative to that of wild-type *Bbre*βgal-III. All variants showed remarkably lower activities than the wild-type (Figure 2B top), and among the variants, R121C displayed the highest relative activity. To determine the transgalactosylation activities, reaction mixtures containing ∼2.5 U/mL of each enzyme were incubated with 600 mM lactose in 50 mM sodium phosphate buffer (NaPB, pH 6.5) at 30 °C for 10 min. When comparing transgalactosylation activities, however, all mutants demonstrated higher activities relative to wild-type *Bbre*βgal-III (Figure 2B bottom). Transgalactosylation activities were determined here as the fraction (or percentage) of global activity that directs toward transgalactosylation during the enzymatic reaction (eq 2 in Materials and Methods). Wild-type *Bbre*βgal-III shows a preference for hydrolysis (70%) over transgalactosylation (30%). When looking at this hydrolysis-to-transgalactosylation ratio of the R121 variants, no clear trend of this ratio for different amino acid-type classification groups could be observed (Figure S3). R121C demonstrated the highest relative global enzyme activity (∼70%) among the variants and also exhibited a relative transgalactosylation activity exceeding 79% (Figure 2B bottom). As all variants exhibit higher relative transgalactosylation activities than the wild type, those variants that display more than 1% relative global enzyme activity were selected for further studies of GOS synthesis. R121L and R121A exhibited comparable global activities, but R121L was selected for further studies due to higher transgalactosylation activity.

We then performed GOS formation in a 24 h lactose conversion and compared the GOS yields by the selected variants and wild-type *Bbre*βgal-III. The GOS yields obtained with selected variants were all higher compared to the yield obtained with wild-type *Bbre*βgal-III (Figure 2C left). This indicates that a substitution of Arg121 by most polar amino acids, and even some nonpolar amino acids such as leucine, isoleucine, and valine, shifts the activity toward transgalactosylation. Wild-type *Bbre*βgal-III reached a maximum GOS yield of 17% of total sugars, whereas the *Bbre*βgal-III variants R121C, R121H, and R121D doubled this yield, reaching 34–39%. The formation and subsequent degradation of GOS during lactose conversion by these three variants are shown in Figure 2C right. It can be seen that GOS are only transiently formed, and they are also subject to “secondary hydrolysis”. The degradation of formed GOS becomes more pronounced when the primary substrate lactose becomes depleted. The maximum GOS yields obtained with these variants showed no significant difference between each other (*p*-value is 0.99). Nevertheless, the two variants R121D and R121H required higher amounts of enzyme (28-fold and 14-fold, respectively, data not shown) due to their low global activities compared to the variant R121C. Therefore, the *Bbre*βgal-III-R121C variant was chosen for further studies.

### Enhanced Formation of β-(1→3)-Linked GOS

The reaction mixtures from lactose conversions by wild-type *Bbre*βgal-III and the *Bbre*βgal-III-R121C variant were subjected to HPAEC-PAD (high-performance anion-exchange chromatography with pulsed amperometric detection) analysis to quantify the individual GOS structures that are formed by the enzymes (Figure S4). It was revealed that the *Bbre*βgal-III-R121C variant produced considerably higher amounts of all formed GOS structures compared to the wild type (Figure 3A–C). Wild-type *Bbre*βgal-III primarily produced Gal-β-1,6-Lac (6′-galactosyllactose) with its highest yield around ∼40% lactose conversion. The *Bbre*βgal-III-R121C variant not only doubled the total GOS yield but also shifted the main GOS product to Gal-β-1,3-Lac (3′-galactosyllactose), reaching its highest yield of 33 mg/mL at ∼50% lactose conversion. Furthermore, the amount of Gal-β-1,4-Lac formed by R121C was also considerably higher than that by the wild-type enzyme. After 50% lactose conversion, Gal-β-1,3-Lac was subject to hydrolysis by R121C, while Gal-β-1,4-Lac and Gal-β-1,6-Lac only started to decrease when lactose was almost completely converted (at 86% and 96%, respectively). Formation of disaccharides was more pronounced after ∼50% lactose conversions by both wild-type and R121C (Figure 3B). The main disaccharides produced by wild-type *Bbre*βgal-III were Gal-β-1,6-Gal (1,6-β-d-galactobiose) and Gal-β-1,6-Glc (allolactose). Transgalactosylation of lactose by R121C also produced Gal-β-1,3-Gal (1,3-β-d-galactobiose) and Gal-β-1,3-Glc; however, β-(1→6)-linked disaccharides were dominant, particularly after 60% lactose conversion, and were hydrolyzed toward the end of the reaction when lactose became depleted. Based on these results, it can be concluded that the R121C mutation in *Bbre*βgal-III not only enhances the total GOS yield but also shifts the regioselectivity toward the formation of β-(1→3)-linked GOS products.

**Figure 3:**
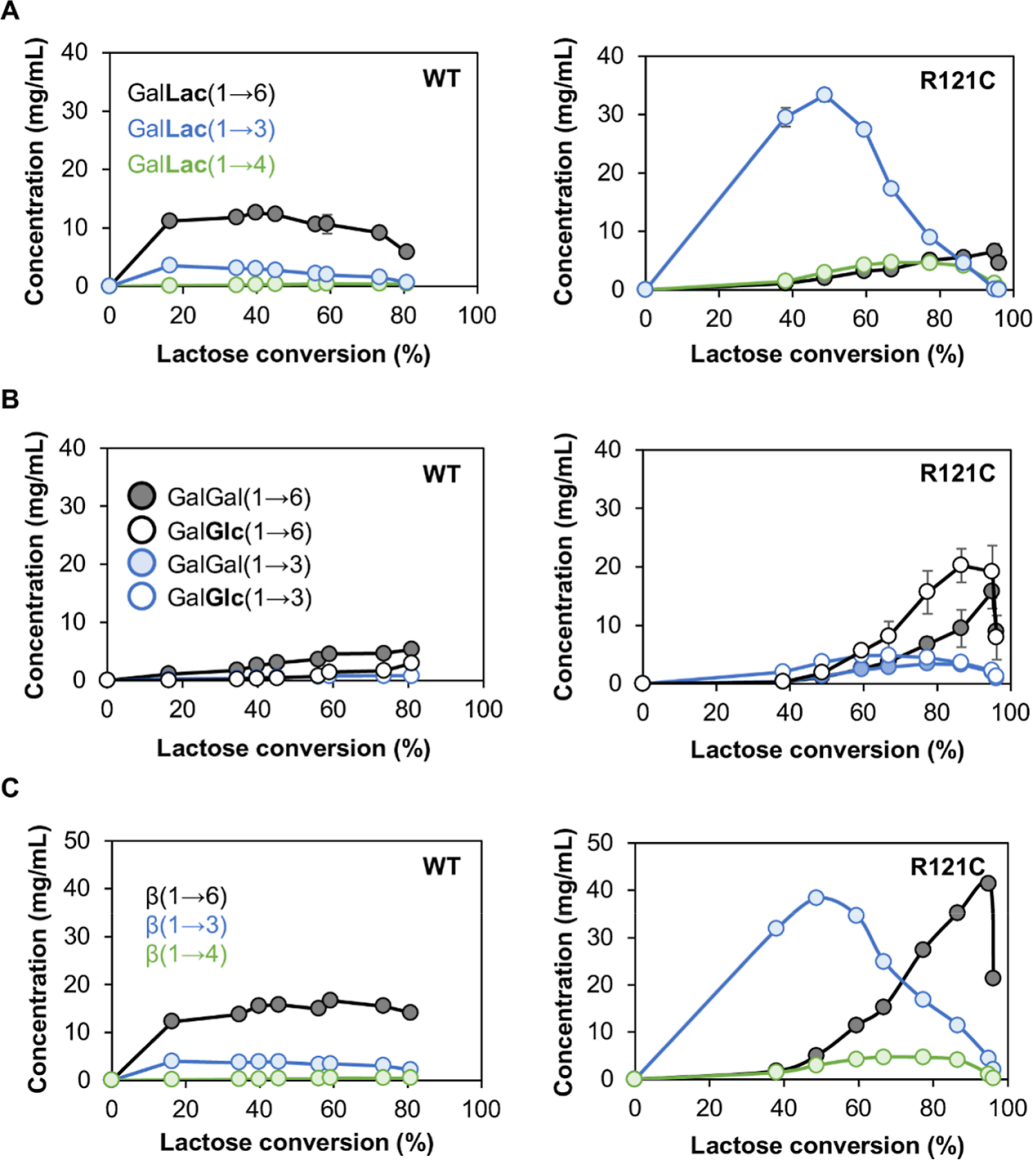
Formation and degradation of individual GOS formed by wild-type *Bbre*βgal-III and the variant *Bbre*βgal-III-R121C during lactose conversion. Reaction conditions were: initial lactose concentration of 600 mM in 50 mM NaPB (pH 6.5) at 37 °C for 24 h and 2.5 U_Lac_/mL of *Bbre*βgal-III (WT) or *Bbre*βgal-III-R121C (R121C). Individual GOS components were identified and quantitated by HPAEC-PAD using authentic GOS as external standards. (A) Trisaccharides; (B) disaccharides; (C) β-(1→6)-, β-(1→4)-, β-(1→3)-linked GOS.

### GOS Size Distribution and Formation of β-(1→2)-Linked GOS

A size distribution analysis of the GOS mixture produced by *Bbre*βgal-III-R121C was performed by using HPSEC-UV (high-performance size exclusion chromatography with UV-based detection). It revealed a broad spectrum of GOS components with a degree of polymerization (DP) ranging from DP2 to DP5 (Figure S5). DP2 and DP3 are the main GOS constituents, and pentasaccharides (DP5) were detected only after 6 h of reaction (∼80% lactose conversion). The concentrations of the various GOS components (DP2 to DP5) decreased toward the end of the reaction (after 48 h at ∼90% lactose conversion) as hydrolysis prevailed over transgalactosylation, since GOS themselves are subject to “secondary hydrolysis”.

The separated fractions representing the di, tri-, tetra-, and pentasaccharide fractions were further analyzed by NMR. Reporter signals of these complex mixtures were compared with extensive literature data^7,32,33^ and cross-checked via ^1^H-^13^C HSQC, COSY, and HMBC experiments for plausibility. The main GOS components identified by HPAEC mentioned above were also supported by one-dimensional ^1^H NMR spectroscopy data.

In the one-dimensional ^1^H NMR spectra, the presence of β-(1→2)-linked GOS including Gal-β-1,2-Glc, which could not be identified by HPAEC-PAD, was clearly confirmed by the presence of peaks, which are significantly shifted downfield to 5.40–5.49 ppm (Figure S6A) while showing HSQC correlations to carbons around 92 ppm (data not shown), which is typical for α anomeric signals of a reducing end. This pattern was found throughout all fractions from the di- to the pentasaccharide levels (Figures S6 and S7). In the disaccharide fraction, reporter signals for all possible combinations of βGal→Gal/Glu were detected (Figure S6A). Herein, at 5.44 ppm, the α anomeric signal corresponding to Gal-β-1,2-Glc was found and an additional signal at 5.48 was also detected, which we suspected could be Gal-β-1,2-Gal.^34^ Furthermore, traces of Gal-β-1,4-Gal could be detected as indicated by the α anomeric signal at 5.268 ppm, while it was not detectable by HPAEC-PAD.

In the trisaccharide fraction, the presence of the 1–2 linkages was also clearly detectable, whereas based on HSQC correlations, we could detect at least four different signals corresponding to different isomers in this region (data not shown). When compared to the wild type, the signal intensity for the 1–2 linkages was notably increased in the trisaccharide fraction of the GOS mixture produced by the *Bbre*βgal-III-R121C (Figure S6B), although we could not unambiguously confirm the exact substitution such as a linear or double substituted pattern of the 1–2 linked species. In the tetra- and pentasaccharide fractions, we could also see a preferred intensity increase of the signal region at 4.2 ppm, which can be attributed to overlapping signals Gal H-4 signal of β-d-Gal*p*-(1→-3)-β-d-Gal*p*- and H-4 signal of β-d-Gal*p*-(1→4)-β-d-Gal*p*- (Figure S7). A further analysis of the ^13^C shifts of this region via HSQC (data not shown) demonstrated an increase in the signal region of 69 ppm, which would reflect an increase for 1–3 type of linkages in this region.^7,32,33^ Both tetra- and pentasaccharide fractions were analyzed by MALDI-TOF to confirm the presence of tetra- and pentasaccharides (Figure S7).

### Specificity toward Various Galactosides

Activities of wild-type *Bbre*βgal-III and the variant *Bbre*βgal-III-R121C with various galactosides are shown as the relative hydrolysis (or conversion) of each substrate in comparison to lactose (Figure 4). Wild-type *Bbre*βgal-III preferentially cleaves β-(1→3)-linked GOS with approximately 5-fold higher rates in hydrolysis (or conversion) compared to lactose. It also exhibited high specificities toward other galactosides, including Gal-β-1,6-Lac, Gal-β-1,6-Gal, and Gal-β-1,4-Lac. Both *Bbre*βgal-III and R121C showed low preferences for Gal-β-1,4-Glc (lactose) and Gal-β-1,4-Gal. The substitution of Arg121 to Cys121 altered the substrate specificity of *Bbre*βgal-III, increasing its preference for Gal-β-1,3-Glc, while remarkably reducing its preferences for Gal-β-1,3-Gal, Gal-β-1,4-Gal, Gal-β-1,4-Lac, and β-(1→6)-linked GOS. Therefore, Arg121 seems to be essential regarding the preferences of the wild-type *Bbre*βgal-III in hydrolyzing β-(1→6)-linked GOS and the 1,3-β-, 1,4-β-, and 1,6-β-d-galactobioses.

**Figure 4:**
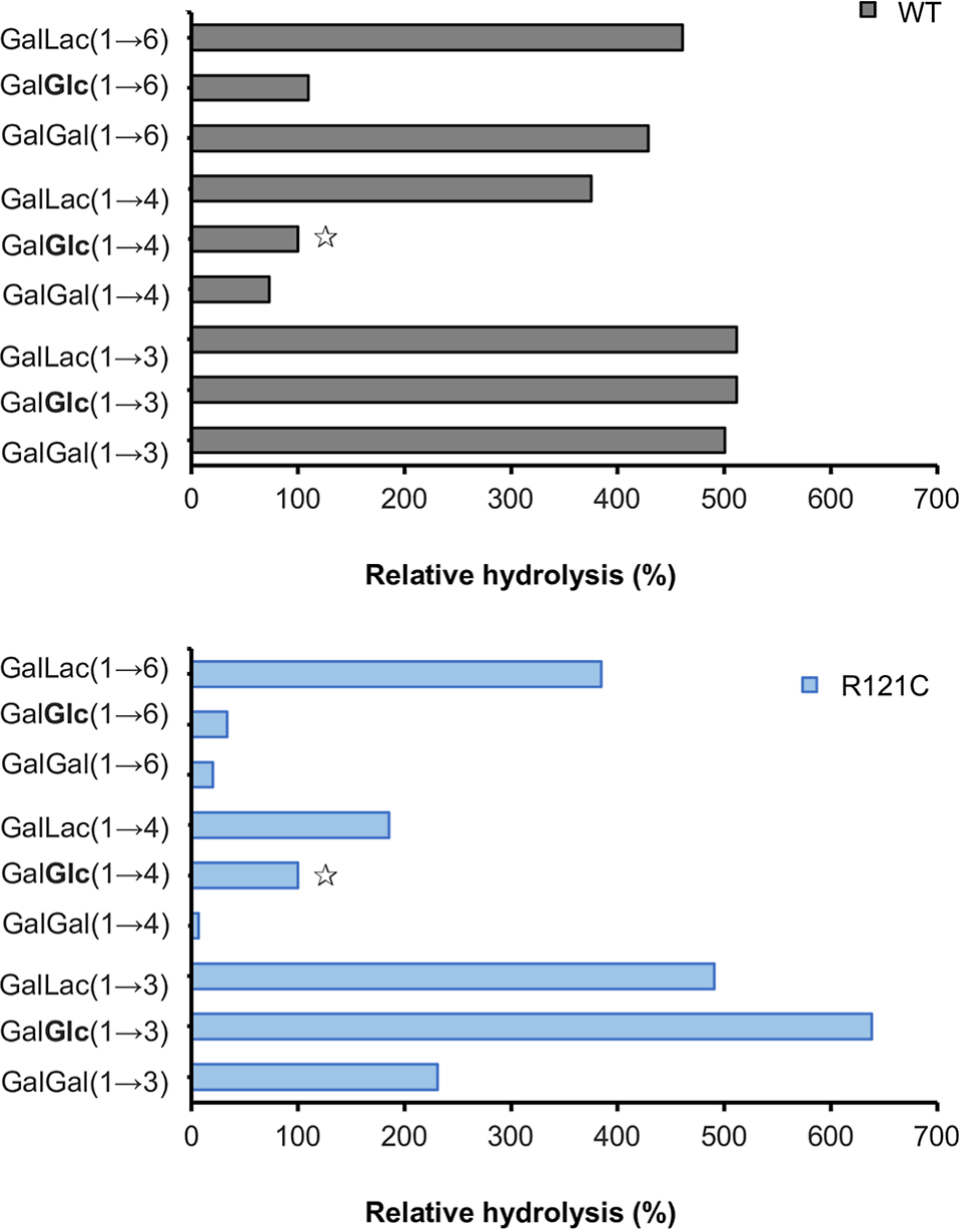
Hydrolysis of different galactosides by wild-type *Bbre*βgal-III (WT) and the variant *Bbre*βgal-III-R121C. The rates of the hydrolysis of each galactoside with an initial concentration of 1 mg/mL using 0.1 U_Lac_/mL was determined at 30 °C and expressed relatively to the hydrolysis of lactose (GalGlc(1→4) indicated with a star open, as 100%). The remaining concentration of each galactoside after 30 min of conversion was quantitated by HPAEC-PAD using authentic GOS as external standards.

### Influence of Reaction Parameters on Transgalactosylation Activity

The ratio [Glc]/[Gal] can be seen as a measure for transgalactosylation and was used to study the influence of varying reaction parameters during lactose conversion on the transferase activity of the variant *Bbre*βgal-III-R121C in comparison with the wild-type *Bbre*βgal-III. The higher the ratio [Glc]/[Gal], the higher transgalactosylation over hydrolysis is expected as it indicates a higher transfer of the galactosyl moieties onto the galactosyl acceptor.

The transferase activity ([Glc]/[Gal] ratio) of the R121C mutant increased with increasing enzyme concentration (Figure 5A) and reached a maximum at ∼5.6 with an enzyme amount up to 60 U/mL. On the other hand, wild-type *Bbre*βgal-III exhibited a maximum [Glc]/[Gal] ratio of 2.2 at an enzyme dosage of 7.5 U/mL and gradually declined when higher enzyme concentrations were used.

**Figure 5:**
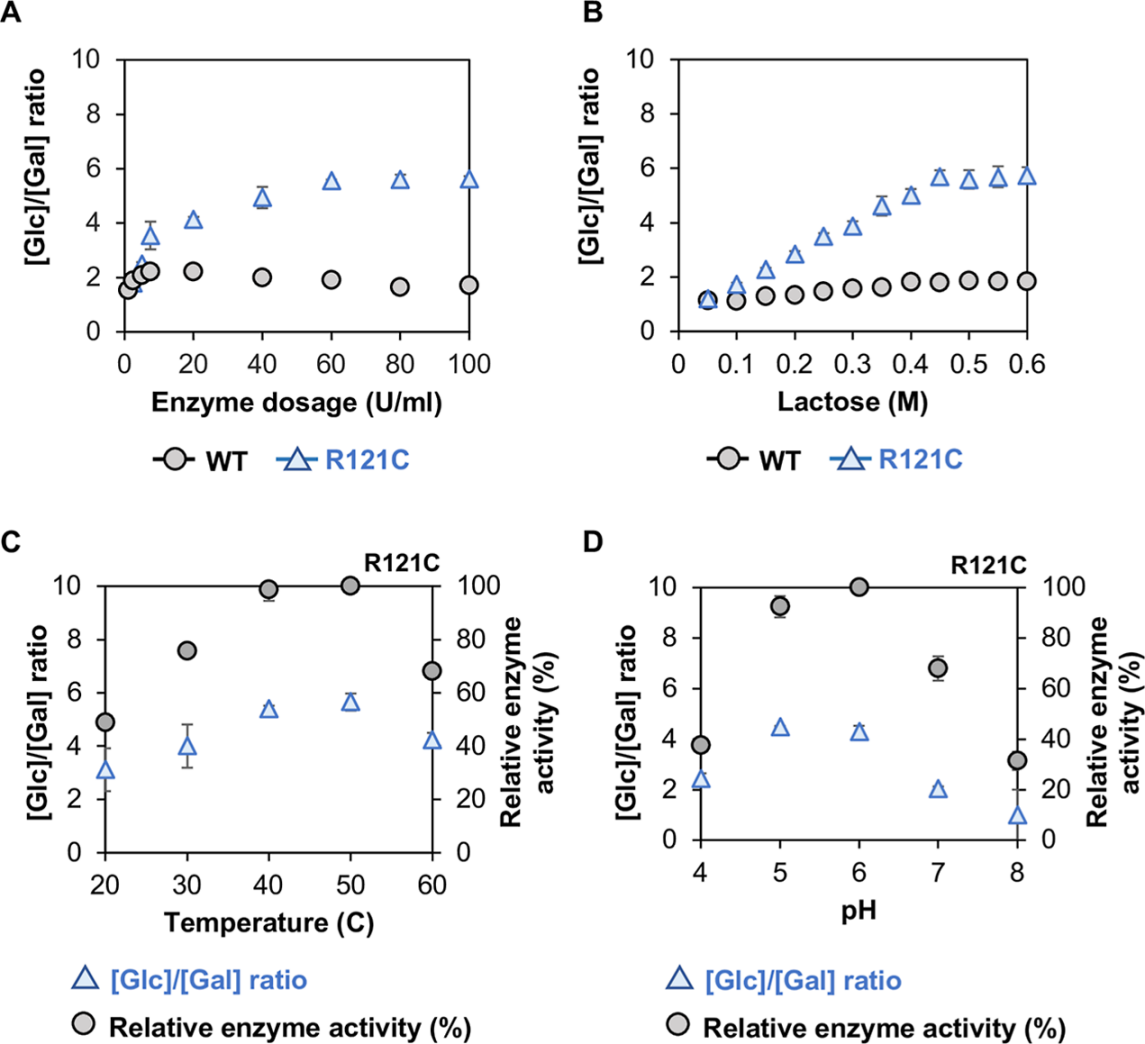
Dependency of the [Glc]/[Gal] ratio and enzyme activity of the variant *Bbre*βgal-III R121C on various factors and incubating conditions (Glc: glucose; Gal: galactose). (A) Enzyme dosage; glucose and galactose released were measured from the reaction mixtures with 600 mM initial lactose in 50 mM NaPB (pH 6.5) at 30 °C and varying amounts of enzyme in the reaction mixture (1–100 U/mL) after 10 min. (B) Substrate concentration; glucose and galactose released were measured from the reaction mixtures with varying concentrations of lactose ranging between 0.05 and 0.6 M in 50 mM NaPB (pH 6.5) at 30 °C after 10 min. (C) Temperature; glucose and galactose released, as well as enzyme activities were measured by the standard assay with 600 mM lactose in 50 mM NaPB (pH 6.5) at varying temperatures between 20 and 60 °C after 10 min. (D) pH; glucose and galactose released, as well as enzyme activities were measured by the standard assay with 600 mM lactose in different buffers, 50 mM phosphate citrate for pH 3–5 or 50 mM potassium phosphate buffer for pH 6–8 at 30 °C, after 10 min.

We also investigated the effect of varying concentrations of lactose on transgalactosylation (or transferase activity) catalyzed by both wild-type *Bbre*βgal-III and R121C (Figure 5B). Low transgalactosylation by wild-type *Bbre*βgal-III was observed over the range of lactose concentrations between 0.05 and 0.6 M as indicated by the [Glc]/[Gal] ratios, which were slightly higher than 1. In contrast, the transferase activity of R121C considerably increased with increasing lactose concentration until it reached a maximum at 0.45 M. These observations with wild-type *Bbre*βgal-III and R121C indicates that the presence of Arg121 in the active site of *Bbre*βgal-III makes the wild-type enzyme consistently favoring water over a sugar galactosyl acceptor, independent of lactose concentrations.

Thermal inactivation (*T*_50_ values) of wild-type *Bbre*βgal-III and R121C was found to be comparable (56 and 53 °C, respectively) (data not shown), suggesting that the mutation affects enzyme activity rather than enzyme stability. The effects of the temperature and pH on transgalactosylation as well as on the activity of R121C were also evaluated (Figure 5C,D). The optimal temperature for R121C activity is between 40 and 50 °C, while the optimal pH is between pH 5 and 6. The highest [Glc]/[Gal] ratios, i.e., the highest transferase activity of R121C, were also found in these temperature and pH ranges, at which the enzyme activity is optimal, as expected. However, R121C exhibited instability above 40 °C upon extended incubation for longer than 1 h (data not shown).

Kinetic parameters of wild-type *Bbre*βgal-III and the variant *Bbre*βgal-III-R121C were measured for the hydrolysis of lactose (Table 1). Steady-state kinetic measurements were performed with lactose as the substrate at concentrations ranging from 50 to 800 mM in 50 mM NaPB (pH 6.5) at 30 °C for 10 min. The concentration of d-glucose in the reaction mixture was measured using the d-Glucose Assay Kit (GOPOD Format) from Megazyme. Looking at these kinetic parameters, the wild-type enzyme displayed a considerably more favorable Michaelis constant when compared to that of R121C (10-fold lower apparent *K*_m_ value). Additionally, the mutation also led to a 2-fold decrease in the enzyme’s turnover number *k*_cat_. Consequently, R121C showed an 18-fold decrease in the catalytic efficiency *k*_cat_/*K*_m_ in comparison to that of the wild-type enzyme.

**Table 1:** Kinetic Parameters of Wild-Type *Bbre*βgal-III (WT) and the Variant *Bbre*βgal-III-R121C for the Hydrolysis of Lactose^a^ ^,^ ^b^

	Michaelis-Menten kinetics		
enzyme	*K*_m_ (mM)	*k*_cat_ (s^–1^)	*k*_cat_/*K*_m_ (s^–1^ mM^–1^)
WT	30 ± 3	85 ± 5	2.8 ± 0.2
R121C	316 ± 7	46 ± 4	0.150 ± 0.002

a The concentration of d-glucose in the reaction mixture was measured using the d-Glucose Assay Kit (GOPOD Format) from Megazyme.

b Steady-state kinetic measurements were performed with lactose as the substrate at concentrations ranging from 50 to 800 mM in 50 mM NaPB (pH 6.5) at 30 °C for 10 min.

### Disruption of the Hydrogen Bond Network in *Bbre*βgal-III-R121C

Biochemical characterization of the variant *Bbre*βgal-III-R121C confirmed that Arg121 plays a crucial role in the hydrolytic activity of wild-type *Bbre*βgal-III. Structural analysis of the predicted model of wild-type *Bbre*βgal-III, which was superpositioned onto the protein templates *Bi*Bga42A (PDB: 8IBT) and BbgII (PDB: 4UCF), revealed that the guanidino group of Arg121 is capable of forming multiple hydrogen bonds. These interactions involve (1) the catalytic Glu160 (the acid/base residue), (2) a water molecule, (3) the glycosidic linkage in the ligand, and (4) the galactose moiety of the ligand (Figure 6A). Interestingly, a shared water molecule between Arg121 and Glu160 is also found in different crystal structures, highlighting the important role of these residues in capturing water. Molecular dynamics simulations support these findings, showing a high occurrence of hydrogen bonds involving the Arg121 residue. The average number of hydrogen bonds formed by Arg121 amounted to 0.62 to Glu160, 1.25 with water molecules, and 0.12 with lactose, which was the substrate used in the simulation. The hydrogen bonds were formed throughout the whole simulation, although the formation of a hydrogen bond between Arg121 and lactose was observed more sporadically in active-site number 2 (Figure S8). Both Arg121 and Glu160 consistently form hydrogen bonds with water molecules in the active site during the molecular dynamics simulations. Accordingly, we hypothesize that the hydrophilicity of the Arg121 side chain is responsible for coordinating a water molecule near the acid/base residue Glu160, facilitating hydrolysis during the deglycosylation step (Figure 6B). This could result in favoring hydrolysis over transgalactosylation in the wild-type enzyme. R121C lacks this guanidino group (Figure 6C) resulting in the disruption of the hydrogen-bonding network, hence potentially affecting substrate positioning and enzyme kinetics.

**Figure 6:**
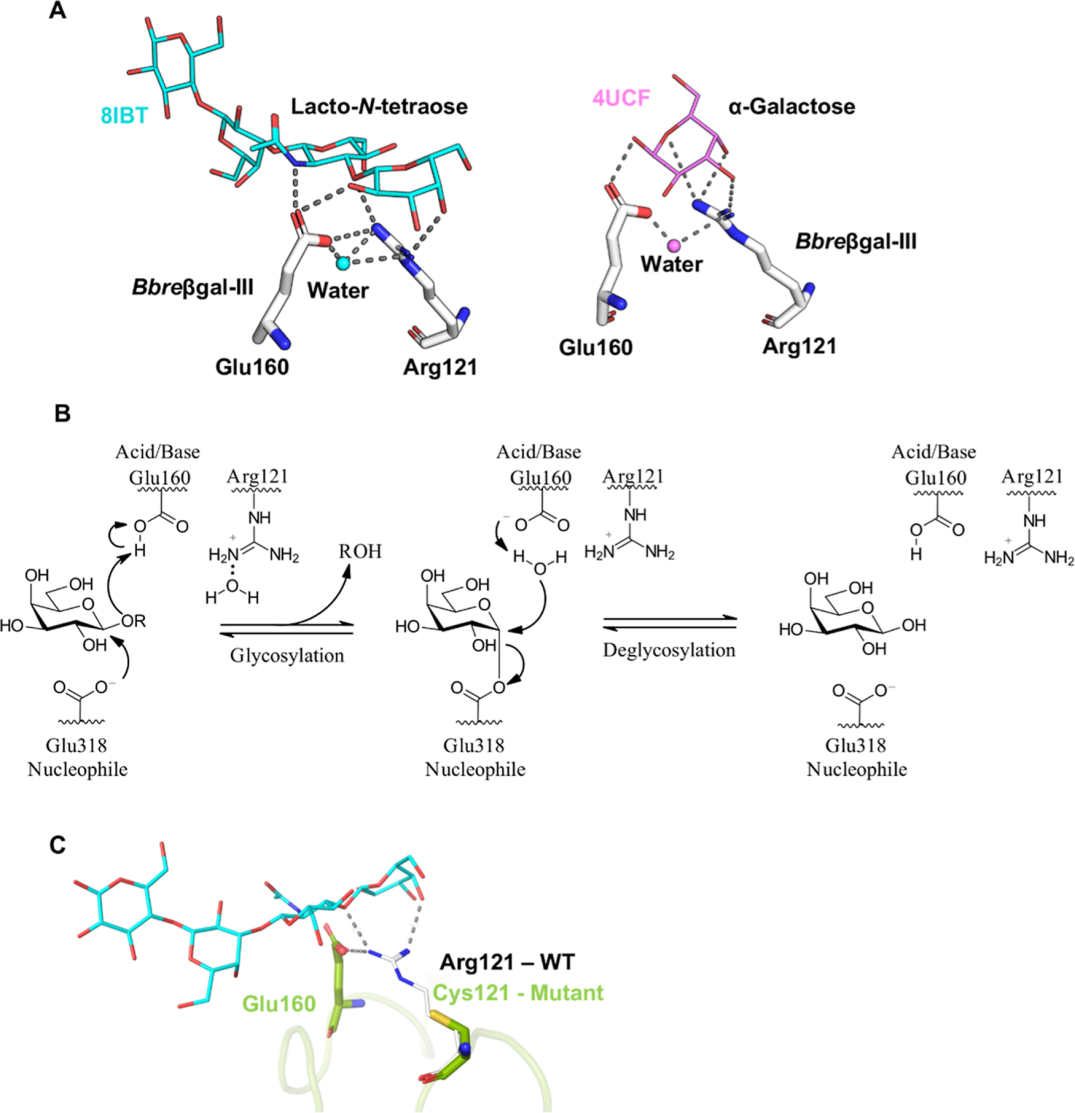
Active-site residue Arg121 and proposed mechanism of *Bbre*βgal-III. (A) Hydrogen bonds between the residue Arg121 and β-d-Gal-(1→3)-β-d-GlcNAc-(1→3)-β-d-Gal-(1→4)-d-Glc (lacto-*N*-tetraose, *Bi*Bga42A (PDB: 8IBT) as template, cyan, left) or α-galactose (BbgII (PDB: 4UCF) as template, pink, right) as ligand, water, and the acid/base residue Glu160 in *Bbre*βgal-III. (B) Proposed catalytic mechanism of the wild-type *Bbre*βgal-III, in which Arg121 coordinates a water molecule favoring hydrolysis over transgalactosylation. (C) Overlay structures of protein loops of *Bbre*βgal-III-R121C (green) and the wild-type *Bbre*βgal-III (WT; gray). Arg 121, the mutated residue Cys121, and the acid/base residue Glu160 are shown in stick representation. The absence of hydrogen bonding between the substrate lacto-*N*-tetraose (cyan) and the acid/base residue Glu160 in the variant *Bbre*βgal-III R121C is illustrated.

### Active-Site Architectures of GH42 and GH2 β-Galactosidases

We compared the active-site architecture of *Bbre*βgal-III with that of the GH2 β-galactosidase from *Bacillus circulans* (β-Gal-II, Lactazyme-B, PDB: 7CWD), which is known to have high transgalactosylation activity for GOS biosynthesis and is widely used in the industrial GOS production.^7,8,35^ The arrangement of catalytic amino acids is similar in both enzymes, with two glutamic acid residues functioning as acid/base and nucleophile residues (Figure 7A left and Figure 7B left). The active sites consist mainly of hydrophobic amino acids including tryptophan, phenylalanine, and tyrosine, facilitating sugar binding. The hydrophilic arginine residue (Arg142) responsible for hydrolysis is also present in the GH2 Lactazyme-B from *B. circulans*. The residues in the active site of the GH2 Lactazyme-B (Figure 7B left) are highly conserved among β-galactosidases,^35^ hence also in GH42 *Bbre*βgal-III (Figure 7A left); however, GH2 and GH42 β-galactosidases exhibit distinct transgalactosylation behaviors. GH2 Lactazyme-B from *B. circulans* provided a maximum GOS yield of 63.4% and preferred to form β-(1→4) glycosidic linkages.^35^ Two GH2 β-galactosidases, *Bbre*βgal-I and *Bbre*βgal-II from the same strain *B. breve* DSM 20213 as *Bbre*βgal-III, were found to be well suited for the production of GOS with total GOS yields of 33% and 44% of total sugars, respectively, and have a propensity to synthesize β-(1→6)- and β-(1→3)-linked GOS.^5^ We speculate that the difference in transgalactosylation capacities between these GH2 β-galactosidases and GH42 *Bbre*βgal-III might be attributed to the location of their active sites. In GH2 Lactazyme-B from *B. circulans*, the active site is located on the surface and therefore is more readily accessible for the substrates and potential galactosyl acceptor molecules (Figure 7B right). In contrast, the active site of GH42 *Bbre*βgal-III is deeply buried within its protein structure and connects to the exterior via a long water tunnel (Figure 7A right).

**Figure 7:**
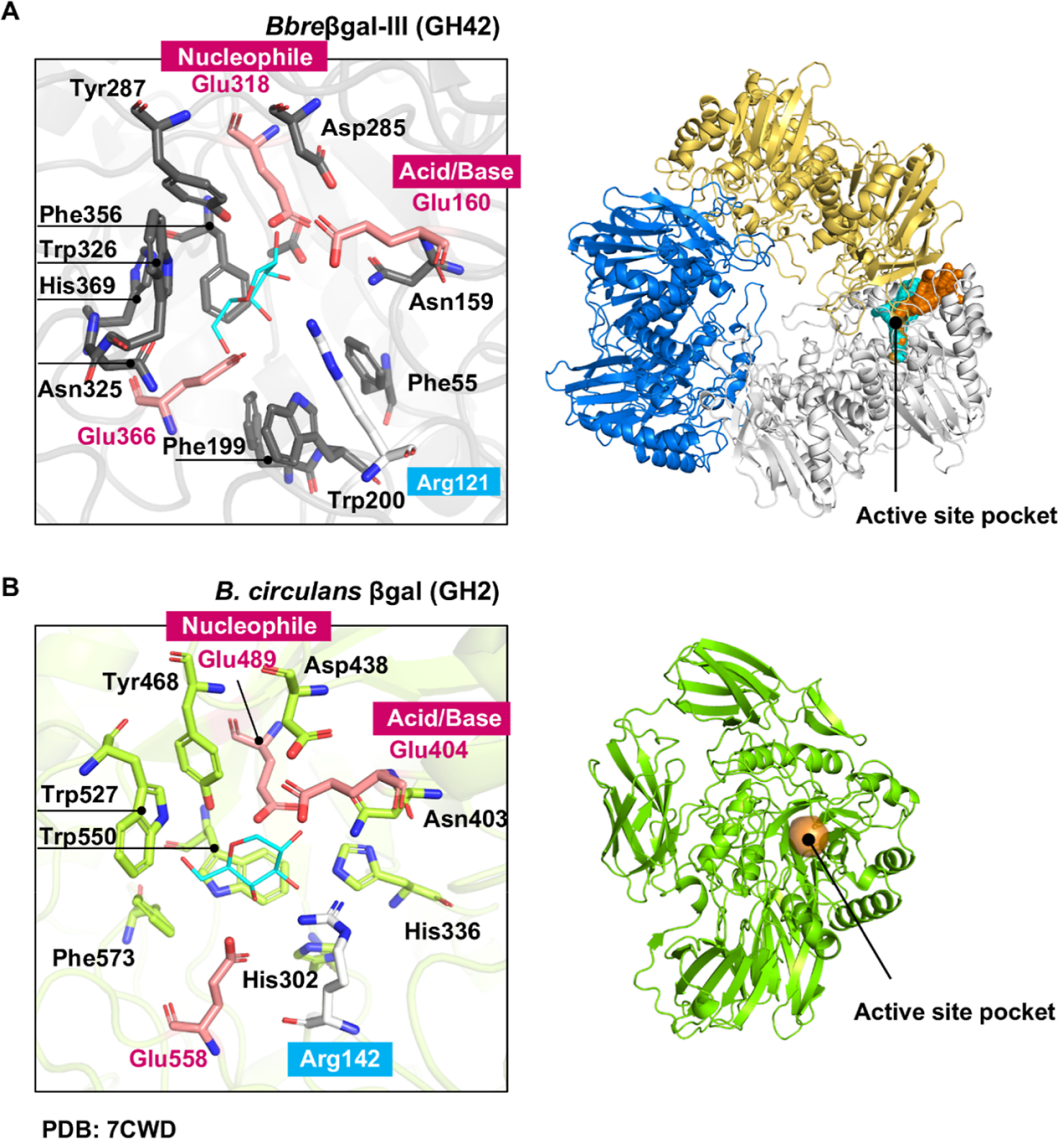
Structural comparison of the active sites of GH42*Bbre*βgal-III from *B. breve* and a representative GH2 β-galactosidase from *B. circulans* ATCC 31382. Residues are shown within 5 Å of the galactose molecule in the active sites of the GH42 *Bbre*βgal-III (A; left) and a GH2 β-galactosidase from *B. circulans* ATCC 31382 (PDB: 7CWD) (B; left). Location of the active sites in the overall structures of the GH42 *Bbre*βgal-III (A; right), showing a deep and elongated tunnel in its trimeric native state (each monomer shown in different color), and the GH2 β-galactosidase from *B. circulans* (B; right), showing a small surface pocket in its monomeric native state.

### Structural Analysis of the Water Tunnels in *Bbre*βgal-III

The analysis using CAVER, a tool for analysis and visualization of tunnels and channels in protein structures, identified two possible tunnels located in domain 1 within the GH42 *Bbre*βgal-III enzyme (Figure 8A). The tunnels are characterized by forking directly after the active site, at the junction located at Phe356 and Arg121. While tunnel A (in light blue) goes into the direction of Ser56 and Phe55, tunnel B (in light orange) goes more into the direction of Gln29 (Figure 8B). The tunnels were detected in 30% and 20% of the simulation time for tunnels A and B, respectively, and were found to be filled with water molecules over the course of the molecular simulation (Figure S9). The number of waters associated with the tunnel gradually increases during the simulation. After approximately 100 ns, the number of water molecules is roughly constant in all three binding sites, suggesting that water does indeed flow into the tunnels. The average number of waters associated with tunnel A was 13.3 and 13.2 for tunnel B after the initial 100 ns.

**Figure 8:**
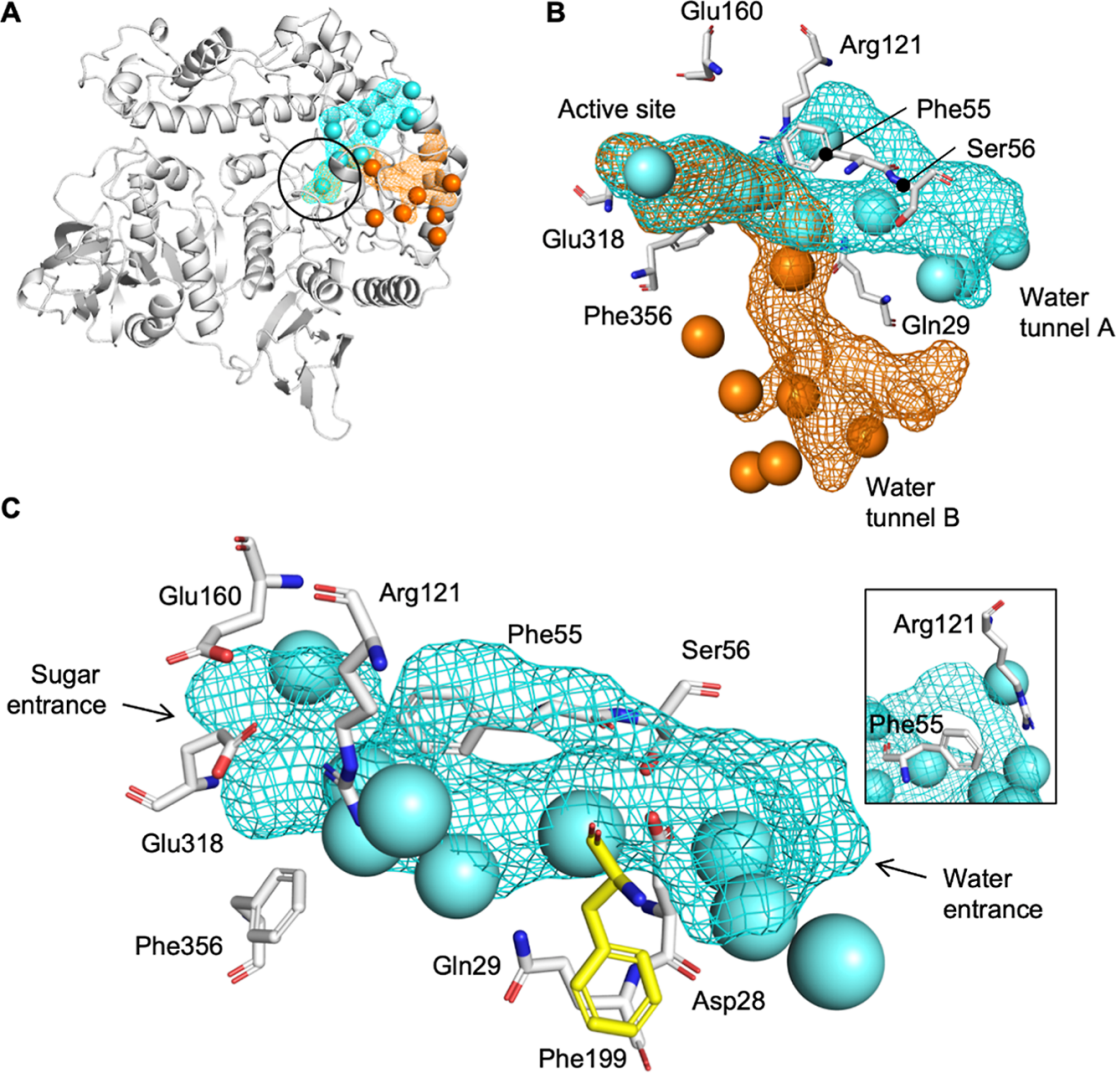
Visualization of water tunnels connecting the active site with the surface of the bulk water. (A) Two water tunnels located in the catalytic domain (domain 1) were found in all monomers of *Bbre*βgal-III. Long buried water tunnels connect the active site (black circle) to the exterior environment. (B) Visualization of the detected tunnels in the molecular dynamics simulation. Tunnel A, colored in light blue, is associated with Phe55 and Ser56. Tunnel B, colored in light orange forks to the other side at the junction described by Phe356 and Arg121, toward Gln29. Tunnel A connects to the acid/base residue Glu160 and is expected to be the main tunnel supplying water molecules to the active site. Phe55, located at the junction between the two tunnels. Visualization of waters associated with the tunnels was based on an arbitrary snapshot of the simulation. Note that some water molecules are not inside the visualized tunnel structure but remain closely associated with the protein surface near the tunnel entrance. (C) Amino acids lining the water tunnel A filled with water molecules. Visualization of waters associated with the tunnel was based on an arbitrary snapshot of the simulation. Phe199, highlighted in yellow, which is an amino acid from an adjacent monomer, indicates that the formation of the water tunnel A depends on the trimeric assembly for proper tunnel formation. The adjacent residues Phe55 and Arg121 are shown in the inset.

Geometrically, water tunnel A is oriented to connect acid/base residue Glu160 within the active-site pocket through Arg121. This long, narrow, and straight tunnel appears to effectively regulate the flow of water molecules from the exterior toward the active site (Figure 8B). On the other hand, water tunnel B has a distal end that opens up more sporadically (Figure S9), which likely hinders the free movement of water molecules toward the catalytic residues.^29^ Moreover, only water tunnel A was observed in the protein templates *Bi*Bga42A (PDB: 8IBT) and BbgII (PDB: 4UCF) and the predicted structure of *Bbre*βgal-III (Figure S10). Therefore, we focused on water tunnel A. Analysis of the radius within the water tunnel A in the initial model reveals several positions of narrow passages that might play a critical role in regulating water passage as a gatekeeper. The opening and closing of the channels were also monitored during the molecular dynamics simulations and observed to be a dynamic process (Figure S9).

First, we aimed to experimentally prove the existence of this predicted water tunnel. The amino acids that form the gatekeeper farthest from the active site were selected for protein engineering (Figure 8C). Alanine scanning mutagenesis was conducted on Asp28, Gln29, and Ser56. Both mutations D28A and Q29A resulted in reducing the enzyme activity by approximately half compared with the wild-type enzyme (Figure 9A). The S56A mutant showed a 2-fold increase in the global enzyme activity compared to wild-type *Bbre*βgal-III. Looking at the residues lining the water tunnel A from the sequence alignment of GH42 β-galactosidases (Figure S11A), Ser56 is the least conserved residues and can be replaced naturally with alanine, which likely widens the water entrance, whereas alanine is not found at other positions (Figure S11B). Therefore, the presence of the water tunnel could be confirmed, as the mutations in the water tunnel crucially affect enzyme activity. Subsequently, we targeted bulkier residues lining the water tunnel, Phe55, Phe199, and Phe356, forming an aromatic-rich region. Alanine scanning results showed a reduction in global enzyme activities with these mutations; however, an increase in transgalactosylation activities was observed for F55A and F199A (Figure 9B).

**Figure 9:**
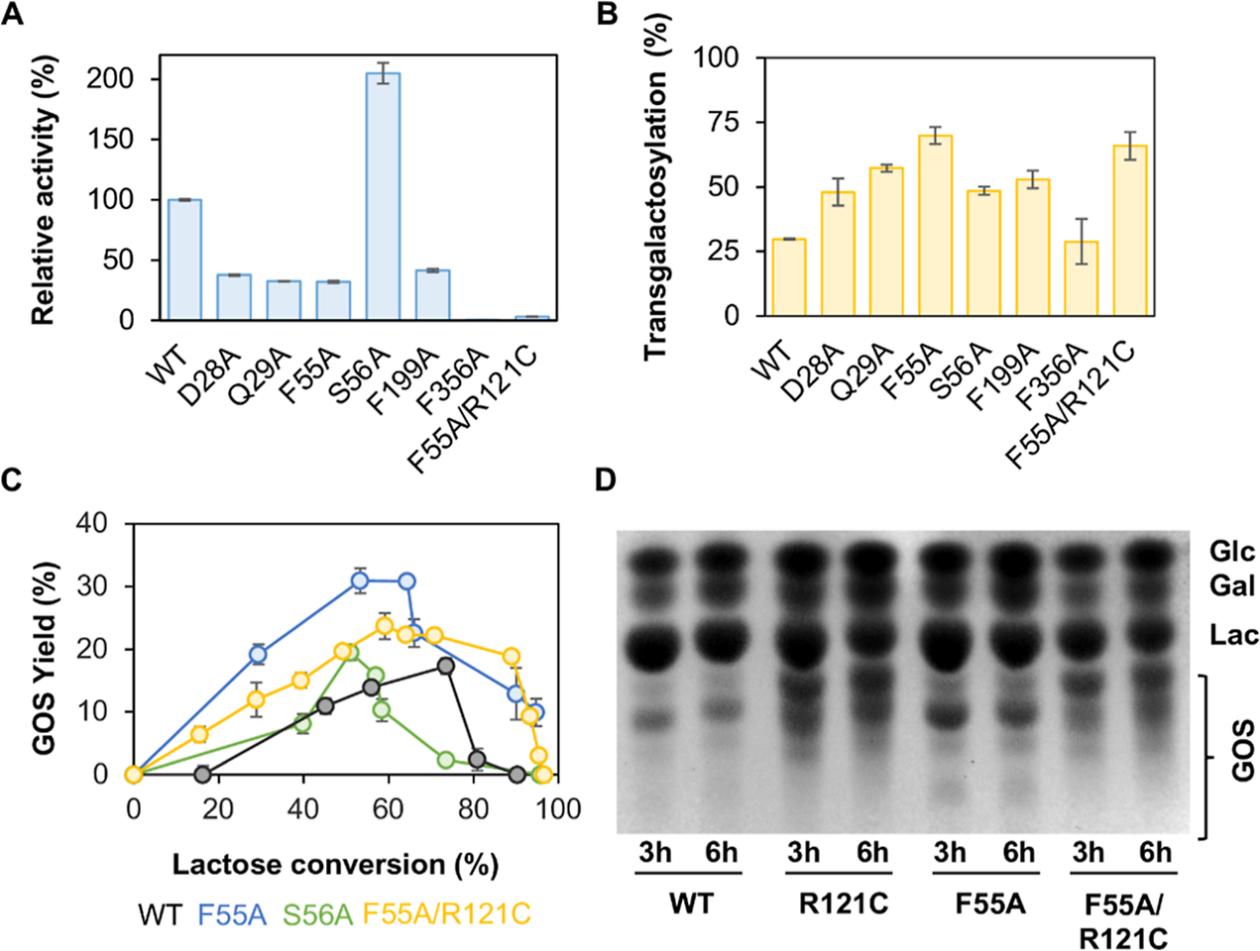
Engineering the water tunnel in *Bbre*βgal-III. (A) Specific enzyme activities (U/mg) given as global activities in the glycosylation step. They were presented as relative enzyme activities to the wild-type (WT). (B) Transgalactosylation activities refer to the direction of E-Gal toward transgalactosylation. (C) GOS yields produced by wild-type *Bbre*βgal-III (WT) and the variants with the mutated residues in the water tunnel. (D) Thin-layer chromatogram of the GOS mixtures after 3 and 6 h of lactose conversion using 600 mM lactose in 50 mM NaPB (pH 6.5) and 37 °C and 2.5 U_Lac_/mL of *Bbre*βgal-III (WT), *Bbre*βgal-III-R121C, *Bbre*βgal-III-F55A, or *Bbre*βgal-III F55A/R121C (right).

Based on the results of alanine scanning, variants F55A and S56A were selected for GOS biosynthesis. A double mutant variant F55A/R121C was also constructed and investigated in transgalactosylation for GOS formation. The results from lactose conversion experiments showed that F55A and F55A/R121C provided 30% and 23% GOS yields, respectively (Figure 9C), whereas R121C could reach a GOS yield of 34% mass of total sugars in the reaction mixture as above-mentioned. The differences in the spectrum of GOS formed during lactose conversion by the wild-type enzyme and these three variants could be clearly seen by thin-layer chromatography (TLC) analysis (Figure 9D). Wild-type *Bbre*βgal-III and F55A had similar patterns of GOS products formed, while the R121C and F55A/R121C variants showed different patterns. These observations were also confirmed by HPAEC-PAD (data not shown). The F55A variant showed an increased transgalactosylation activity leading to an increase in the GOS yield compared to the wild-type, but no change in the main GOS components was observed. On the other hand, the F55A/R121C variant not only improved the GOS yield compared to wild-type but also showed the shift in linkage formation of the main GOS components produced during lactose conversion similarly to R121C. To better understand whether the amino acid substitutions in the water tunnel caused any changes in protein stability, we also observed a considerable decrease in thermal stability (*T*_50_ value) of the F55A variant (48 °C) compared to wild-type *Bbre*βgal-III (56 °C). Therefore, water tunnel engineering affects not only enzyme activity but also its stability.

## Discussion

The GH42 β-galactosidase *Bbre*βgal-III from *B. breve* DSM20213 showed low transgalactosylation activity compared to the two GH2 β-galactosidases (*Bbre*βgal-I and *Bbre*βgal-II) from the same strain.^3,5,27^ To increase the transgalactosylation activity, it was reported that mutations at the sugar donor subsites could shift the reaction of enzymes toward transgalactosylation.^36,37^ The highly conserved residue Arg121, which is at the −1 subsite and the only hydrophilic residue besides the catalytic residues in the active site of *Bbre*βgal-III, was selected for site-saturation mutagenesis. All of the Arg121 variants displayed increased transgalactosylation activities. The variants with smaller amino acid side chains replacing arginine at position 121 likely retained their global enzyme activity with the exception of proline and glycine, which may be unfavorable at this position due to the conformational rigidity of proline and the flexibility of glycine. This observation suggests that bulky amino acids at this position hamper the activity, maybe restricting access of the substrate to the active site. A further analysis of amino acid polarity revealed that most polar substitutions retained their global enzyme activity, whereas most nonpolar substitutions at this position resulted in reduced activity. Due to the low transgalactosylation activity of *Bbre*βgal-III, a low GOS yield of 17% mass of total sugars was obtained. This yield is comparable to the GOS yield of 18% obtained from transgalactosylation of lactose by another GH42 β-galactosidase, BgaB from *B. breve* UCC2003, which shares 99.28% sequence identity with *Bbre*βgal-III.^38^ Site-saturation mutagenesis at Arg121 successfully increased transgalactosylation, and a 2-fold increase in the GOS yield could be obtained with the *Bbre*βgal-III-R121C variant. A study by Placier et al. (2009) also reported that the variant R109W of a GH42 β-galactosidase from *Geobacillus stearothermophilus*, which was obtained through random mutagenesis followed by site-saturation mutagenesis, showed an improvement in the yield of Gal-β-1,3-Lac from 2% with the wild-type enzyme to 23%.^39^ Moreover, structural analysis of Arg109 reveals a direct hydrogen bond interaction with the O3 and O4 positions of the galactose moiety, similar to the interaction observed with Arg121 in this study.

An increase in the formation of all main detected GOS components was observed in the R121C variant. Interestingly, wild-type *Bbre*βgal-III primarily produces Gal-β-1,6-Lac, while R121C produces mainly Gal-β-1,3-Lac in the first half of the reaction up to 50% lactose conversion. Their active-site geometries allow the hydroxyl group at either C3 or C6 of the galactopyranosyl moiety in lactose molecules (here as a sugar acceptor) to be positioned close to the acid/base residue Glu160 before getting deprotonated and linked to galactose at the −1 subsite. This mechanism is part of the deglycosylation step in a retaining double-displacement reaction. The high transgalactosylation activity of the R121C variant enables it to synthesize and accumulate β-(1→3)-linked GOS, mainly Gal-β-1,3-Lac, followed by β-(1→6)- and β-(1→4)-linked products. It is hypothesized that β-galactosidases, which preferentially hydrolyze certain GOS structures, also preferentially form these glycosidic linkages when acting in the transgalactosylation mode. The preference in linkage formation during transgalactosylation catalyzed by *Bbre*βgal-III-R121C supports the above-mentioned hypothesis, as this variant also shows high specificities toward hydrolyzing galactosides with β-(1→3) linkages (Figure 4). However, the accumulation of β-(1→3)-linked GOS, mainly Gal-β-1,3-Lac, is confined to only up to 50% of lactose conversion as the hydrolysis of these GOS components becomes more prominent at higher lactose conversions. Our data also clearly indicate that the R121C variant prefers lactose as the galactosyl acceptor over glucose and galactose, as shown by the dominant formation of the trisaccharide Gal-β-1,3-Lac. Conversely, Gal-β-1,6-Glc (allolactose) and Gal-β-1,6-Gal are not preferred substrates for *Bbre*βgal-III-R121C as shown by low hydrolysis rates compared to other galactosides (Figure 4). Even though the formation of these components was only slow in the first half of the reaction, it increased after 50% lactose conversion. As a result, Gal-β-1,6-Glc and Gal-β-1,6-Gal accumulated during the second phase of transgalactosylation and were only hydrolyzed toward the end of the reaction when lactose was depleted.

The predicted structure of the interaction between the R121C variant and ligands lets us hypothesize how it transitions from a hydrolase to a transglycosylase enzyme. The structural model of the enzyme-substrate complex reveals a hydrogen bond between Arg121 and the catalytic acid/base residue Glu160 (Figure 6A). This hydrogen bond stabilizes the conformation of the acid/base residue, which is essential for its enzymatic activity in both glycosylation and deglycosylation. This is further supported by the pH dependence of the enzyme activity. The R121C mutant shows a notable decrease in activity at pH greater than 7. At alkaline pH, Glu160 is deprotonated and negatively charged, while Arg121 in wild-type *Bbre*βgal-III remains positively charged, forming a salt bridge. In R121C, the absence of this positively charged residue destabilizes Glu160, impairing its proper orientation for catalysis and resulting in reduced activity (Figure S12). Additionally, the smaller thiol side chain of cysteine in R121C, compared to the guanidino group of arginine in wild-type *Bbre*βgal-III, creates an additional space in the active site. Consequently, the increased flexibility of the acid/base residue and the active site in the enlarged −1 subsite slows down the deglycosylation reaction. This allows the galactosyl-enzyme intermediate more time to deprotonate a large sugar molecule instead of water, which eventually becomes the galactosyl acceptor.^40,41^ Furthermore, for hydrolysis to occur, the nucleophilic water molecule needs to be precisely positioned to be deprotonated by the base residue Glu160.^42^ Without assistance through the hydrogen bond network of the hydrophilic residue Arg121, it becomes difficult for the water molecule to reach its catalytically relevant position for deprotonation. The Michaelis–Menten kinetic analysis confirms that the mutation R121C weakens lactose binding in the active site of *Bbre*βgal-III-R121C as shown by a higher *K*_m_ value for lactose, i.e., lower affinity for lactose, compared to the wild-type enzyme.

It was reported that a glycosynthase-like mutant of a GH42 β-galactosidase (Aaβ-gal) from *A. acidocaldarius*, which shows efficient transglycosylation activity, was successfully created by mutating a noncatalytic glutamic acid residue (Glu361Gly).^30^ Comparing the protein sequences between Aaβ-gal and *Bbre*βgal-III, it shows a coverage of 93% and a protein identity of 35.29%. Therefore, Aaβ-gal and *Bbre*βgal-III are not orthologous sequences. However, structural alignment of these proteins gives an RMSD of 1.161, suggesting that the two structures have highly similar folds. Moreover, Glu361, a critical position in Aaβ-gal, forms a hydrogen bond with the hydroxyl group at C4 of the galactose molecule. Similarly, Glu366 in *Bbre*βgal-III, a noncatalytic residue, interacts with the hydroxyl group at C4 of galactose. Our experiments showed that site-saturation mutagenesis at Glu366 leads to enzyme inactivation (data not shown), which is similar to the observation in Aaβ-gal. Therefore, we believe a glycosynthase-like mutant of *Bbre*βgal-III with improved transgalactosylation activity could also be engineered. However, the glycosynthase-like mutant of Aaβ-gal strongly requires sodium azide for its catalysis. We aim to enhance transgalactosylation activity of *Bbre*βgal-III for the improvement in the yield of GOS products for food applications; hence, engineering strategies, which might also require toxic substances, are not favorable, as a safe and nontoxic system should be used for GOS biosynthesis.

In a recent study by Luang et al. 2025, it was demonstrated that GH3 hydrolases employ a structured water-binding network in which noncatalytic residues, such as Glu220 and Lys260, stabilize and orient catalytic water molecules to support hydrolysis.^43^ In agreement with this, our study reveals that similar water-coordinated motifs are also present in our GH42 *Bbre*βgal-III (this study) and the GH2 BgaD-D.^44^ In these enzymes, Arg121 (in GH42 *Bbre*βgal-III) and Arg185 (in GH2 BgaD-D) occupy positions comparable to Lys260 in GH3, suggesting that a conserved hydrophilic residue is required for proper water positioning. Furthermore, both enzymes show a non-nucleophilic glutamate, Glu366 in GH42 *Bbre*βgal-III and Glu601 in GH2 BgaD-D, at which mutations completely abolish enzymatic activity, underscoring its critical role in catalysis. These findings highlight the importance of noncatalytic residues in coordinating structured water networks within the active sites in glycoside hydrolases. We propose that this water-binding motif, consisting of a hydrophilic residue and a noncatalytic glutamate, represents a conserved structural feature across glycoside hydrolase families GH2, GH3, and GH42.

Moreover, Arg121 possibly forms hydrogen bonds with water molecules passing through water tunnel A. The guanidino group in the Arg121 side chain possibly interacts with these water molecules, which could serve as a water reservoir close to the active site and favor the hydrolytic reaction. This explains how the wild-type *Bbre*βgal-III preferentially performs hydrolysis over transgalactosylation, even under high lactose concentrations.

We hypothesize that the highly conserved Arg residue in the vicinity of the acid/base residue, which is found in many GH2 ^35^ and GH42 β-galactosidases,^39^ is a potential switch for transgalactosylation in other β-galactosidases as well. Our experiments showed that the R402A mutation of GH2 *Bbre*βgal-II (NCBI Reference No. EFE88654.1) from *B. breve* DSM 20213, which is also located adjacent to the acid/base residue in the hydrophobic active site, enhanced the transgalactosylation activity for improved *N*-acetyllactosamine synthesis using lactose and *N*-acetylglucosamine as a sugar donor and acceptor, respectively (data not shown). However, mutations at the residue R185 located near the acid/base residue in the active site of the GH2 BgaD from *B. circulans* ATCC31382, including R185E, R185G, R185L, R185P, R185S, and R185 K, made the enzyme completely inactive.^44^ Thus, considering the hydrophilic residue nearby the acid/base residue as a hotspot for mutagenesis to increase transgalactosylation activity in β-galactosidases is still ambiguous and requires further investigation.

Even though the residues in the active sites of GH42 and GH2 β-galactosidases are highly conserved, a key difference in their active-site architectures appears to be the presence of a water tunnel in GH42 β-galactosidases. Our results showed that engineering the water tunnel can increase the transgalactosylation activity. This suggests that modification of the water tunnel alters the movement of internal water molecules, which in turn affects enzyme activity.^29^ In a study by David and colleagues,^29^ two water channels were identified in a GH16 endo-β-agarase AgaD from *Zobellia galactanivorans*, one of which is about 13 Å in length and connects the bulk water to the catalytic acid/base in the active site. Two pairs of residues, Gln342/Tyr181 and Asp341/Ser351, were identified to form two putative bottlenecks at each end of water channel 1, which could act as gatekeepers. Simulation work in this reported study confirmed that alteration of water channel 1 drastically reduced water access to the active site, as water molecules displayed shorter residence times and faster purge dynamics. This limited water availability at the catalytic center shifted the reaction balance toward transglycosylation rather than hydrolysis. Thus, modification of the tunnel altered the internal water flow, which appears to play a critical role in regulating catalytic outcomes. In their wild-type enzyme, water molecules within the tunnel are relatively well structured and persistent, ensuring that the catalytic acid/base residue consistently receives water to perform hydrolysis efficiently. Consequently, mutating any amino acid lining water channel 1 may disrupt the flow of water molecules, as water molecules may no longer be able to pass through the channel or escape more rapidly through the alternative channel instead of remaining near the catalytic site. Mutations at the positions Gln342, Tyr181, Asp341, and Ser351 resulted in mutant enzymes that had lost most of their hydrolytic activity while keeping and enhancing their transglycosylase activity.^29^ Similarly to AgaD, two water tunnels A and B were identified in *Bbre*βgal-III. A water gate formed by Asp28, Gln29, and Ser56 lining tunnel A (Figure 8C) is more than 20 Å from the catalytic residues. We also observed that disruption of the water channel resulted in increased transglycosylation, hence reduced hydrolysis. The Phe55 residue is close to the separation point of channels A and B (Figure 8B), such that the F55A mutation likely influences the water flow through both channels. Removing the bulky aromatic side chain exactly at the joint of the two channels will possibly enhance leakage from channel A into channel B (or vice versa) or lead to a structural disruption that may interfere with the overall enzyme architecture. The average of the shortest distance between any nitrogen of the Arg121 guanidinium group and the ring carbons of Phe55 amounts to 3.8 Å in the MD simulations, suggesting that the removal of Phe55 could affect the position of Arg121 as well.

Our study demonstrates that the enhancement in transgalactosylation of GH42 *Bbre*βgal-III from *B. breve* can be achieved by both active site and water tunnel engineering. The disruption of the hydrogen bond network in the active site of the *Bbre*βgal-III-R121C variant completely alters enzyme properties in the transgalactosylation reaction such as substrate specificity, sugar acceptor preference, and linkage formation of the main GOS components. On the other hand, water tunnel engineering affects water dynamics but retains preference of product formation of the wild-type also in the variant, as seen in *Bbre*βgal-III-F55A. Therefore, different protein engineering strategies can lead to the formation of GOS mixtures with different compositions.

## Conclusion

This study provides a comprehensive investigation into factors influencing the low transgalactosylation activity in the GH42 β-galactosidase family and reveals a key residue facilitating hydrolysis. The active-site architecture of GH42 β-galactosidases with its deeply buried active site connected to an internal water tunnel is very different from that of GH2 β-galactosidases, which possess high transgalactosylation activity and surface-exposed active sites. This distinct active-site architecture necessitates a hydrogen bond network through the arginine residue, favoring hydrolysis in GH42 β-galactosidases. We also demonstrated various engineering strategies both to improve transgalactosylation yields and to alter substrate sugar preference of *Bbre*βgal-III from *B. breve* DSM 20213, a GH42 β-galactosidase. The structure–function insights gained in this work could be advantageous for engineering novel enzymes with desirable functions for the biosynthesis of glycans.

## Materials and Methods

### Structural Analysis

The gene encoding *Bbre*βgal-III (accession no BAQ99491.1) from *B. breve* DSM 20213 (GenBank genome sequence AP012324.1) was used to predict the oligomeric structure by AlphaFold3.^31^ The active site of the enzyme was identified using PyMOL software (version 2.4)^45^ by overlaying it onto an experimental structure in the PDB database (8IBT with a 95.37% protein identity). The amino acid alignment with other 301 GH42 β-galactosidases available in the Uniprot database was performed using MAFFT^46^ (retrieved in November 2025). Promising residues were then selected for further experiments.

### Molecular Dynamics Simulations

Molecular simulations were started from an earlier AlphaFold2 model and superposed with the crystal structure of GH42 BbgII from *B. bifidum* S17 (PDB: 4UCF). The binding site was identified based on the position of the galactose unit in this reference structure. The quickprep module of the Molecular Operating Environment^47^ was used to determine the protonation state of all residues and to dock lactose as a substrate into the binding site. The highest scoring binding pose was subsequently superposed into all three binding sites of *Bbre*βgal-III. The model was subsequently simulated using the openMM^48^ molecular dynamics software (version 8.2). The protein was parameterized with the AMBER ff14SB^49^ and the ligand using the GLYCAM_06j-1 ^50^ force field. A nonbonded cutoff of 1.0 nm was used, and long-range electrostatics were calculated using PME. All simulations were run at 300 K, using a Langevin middle integrator^51^ with a timestep of 2 fs and a friction coefficient of 1 ps^–1^. After an energy minimization in vacuum, the complex was solvated in a cubic box with a 14.562 nm edge length using 87157 TIP3P water molecules together with 361 sodium and 237 chloride ions to neutralize the net charge of the system and to set the ionic strength to approximately 150 mM. A subsequent energy minimization was done, followed by a 1 ns simulation at a constant volume. Afterward, a 500 ns simulation under constant pressure at 1 bar was carried out using a Monte Carlo barostat.

Hydrogen bond analysis was done using MDAnalysis^52–54^ (version 2.9.0) on snapshots that were taken every 10 ps during the simulation. For the calculation of the average number of hydrogen bonds, the first 100 ns of the simulation was removed for equilibration. The detected hydrogen bonds were aggregated for each interacting residue pair, and the mean occupancy across the three active sites was reported.

The analysis of the water tunnels was carried out using CAVER^55,56^ (version 3.03 BETA) on snapshots taken every 10 ns during the simulation. Before the CAVER analysis was run, the frames were aligned on the C-alpha atoms using MDAnalysis. CAVER was run with the following settings: the starting point was set to the E318 residue, the probe radius to 0.9 Å, the shell depth to 4 Å, the shell radius to 3 Å, the desired radius to 5 Å, the minimum middle zone to 10 Å, the minimum tunnel length to 15 Å, the starting point protection radius to 10 Å, the maximum distance to 3 Å, and the clustering threshold to 5 Å. Detected tunnel clusters were subsequently visualized using PyMOL (version 3.1.0) and refined manually by only selecting those that go from the active site outward to the protein surface.

The quantification of waters in the tunnels was done using Python and MDAnalysis, counting the number of waters in every snapshot where the oxygen was within 2 Å of any tunnel lining atom of the clusters as reported by CAVER. While this approach will also count waters that are close to the tunnel entrance but not necessarily inside the tunnel, visual inspection has shown that the majority of the waters detected were inside tunnels.

### PCR-Based Mutagenesis at the Active Site and in the Water Tunnel

The expression plasmid pET21a­(+) containing the *Bbre*βgal-III gene was used as a template. Primer pairs containing the overlapping and extended regions were used as indicated (Table S1). The PCR reaction mixture contained Q5 High-Fidelity DNA Polymerase, nucleotides, Mg^2+^, primers, and DMSO. A two-step PCR was conducted in a thermal cycler with denaturation and extension using the following condition: 98 and 72 °C for 10 s and 3.5 min, respectively. PCR products were purified using the DNA Gel Extraction Kit (New England Biolabs), followed by *Dpn*I digestion before being transformed into *E. coli* NEB5α and BL21 (DE3). Transformed cells were plated onto LB agar plates containing 100 μg/mL of ampicillin and incubated at 37 °C overnight. Mutations were verified by sequencing.

### Gene Expression and Protein Purification

Successful transformants of *E. coli* BL21 (DE3) were cultured in 400 mL of LB medium with 100 μg/mL of ampicillin at 37 °C for 12 h. When the OD_600_ reached a value of 0.6, 0.1 mM IPTG was added and the incubation continued at 20 °C for 12 h. Bacterial cells were collected by centrifugation and disrupted using a sonicator (Bandelin Sonopuls HD60, Berlin, Germany). The resulting cell-free crude extracts were obtained through high-speed centrifugation (35,000*g* for 30 min, 4 °C). Protein concentration was determined using Bradford’s reagent.^57^ Crude cell extracts were subjected to protein purification using a 5 mL HisTrap HP Ni-immobilized metal ion affinity chromatography column (Cytiva, MA, USA). The crude enzyme solution was loaded onto the column and washed, and the His-tagged protein was eluted with a gradient of buffer A (20 mM sodium phosphate buffer [NaPB], 20 mM imidazole, 500 mM NaCl, pH 6.5) and buffer B (20 mM NaPB, 500 mM imidazole, 500 mM NaCl, pH 6.5) from 0 to 100% of buffer B. Protein fractions exhibiting an absorbance at 280 nm were collected and concentrated using an Amicon Ultra Centrifugal Filter Unit with a 30 kDa cutoff membrane (Millipore, MA, USA). The protein purity was determined by SDS-PAGE.

### Determination of Enzyme Activity

#### Global Enzyme Activity

The overall or global β-galactosidase activity, which is defined by the initial release of glucose from the enzyme-substrate complex, was determined using lactose as a substrate. A 600 mM lactose solution in 50 mM NaPB, pH 6.5 (480 μL) was mixed with 20 μL of enzyme and incubated at 30 °C and 600 rpm for 10 min. After the reaction was stopped at 95 °C for 10 min, the release of d-glucose was measured using the d-Glucose Assay Kit (GOPOD Format) from Megazyme (Bray, Ireland), in which the concentration of d-glucose was determined using a GOD/POD (glucose oxidase/peroxidase) assay. One unit of global activity was defined as the amount of β-galactosidase releasing 1 μmol of d-glucose per minute under the specified conditions. The specific activity was calculated using the following equation:

1 $$specific\;activity\;\left(U/mg\right)=\frac{enzyme\;activity\;\left(U/mL\right)}{protein\;concentration\;\left(mg/mL\right)}$$

#### Transgalactosylation Activities in the Deglycosylation Step

To determine transgalactosylation activities, a reaction mixture containing ∼2.5 U/mL of enzyme was incubated with 600 mM lactose in 50 mM NaPB (pH 6.5) at 30 °C for 10 min. The reaction was stopped by heating at 95 °C for 10 min. The concentrations of d-glucose and d-galactose in the reaction mixture were measured using the d-Glucose Assay Kit (GOPOD Format) and l-Arabinose/d-Galactose Assay Kit, respectively, from Megazyme. The transgalactosylation activities in the deglycosylation step were calculated using the following equation:

2 $$transgalactosylation\;\left(\%\right)=\frac{\left[Glc\right]-\left[Gal\right]}{\left[Glc\right]}\times 100$$

with [Glc]: glucose concentration (mg/mL); [Gal]: galactose concentration (mg/mL)

### Steady-State Kinetic Measurements

Steady-state kinetic parameters, including *K*_m_, *k*_cat_, and *k*_cat_/*K*_m_, were measured as global enzyme activities using lactose as the substrate. Enzyme (20 μL) was incubated with 480 μL of lactose solution with varying concentrations (50–800 mM) in 50 mM NaPB (pH 6.5) at 30 °C for 10 min. The reaction was stopped by heating at 95 °C for 10 min. The concentration of d-glucose in the reaction mixture was measured using the d-Glucose Assay Kit (GOPOD Format) from Megazyme. The determination of the kinetic constants and parameters was performed using the one-site saturation equation in SigmaPlot 14.0 (SPSS Inc., Chicago, IL).

### Biosynthesis of GOS

A reaction mixture for lactose conversion contained 2.5 U/mL of enzyme, and 600 mM lactose was incubated at 37 °C for 24 h with agitation at 180 rpm. Samples were taken at regular time intervals and immediately heated at 95 °C for 10 min to stop the enzymatic reaction. d-Glucose, d-galactose, and d-lactose were measured using the d-Glucose Assay Kit (GOPOD Format), the l-Arabinose/d-Galactose Assay Kit, and the Lactose Assay Kit - Sequential/High Sensitivity, respectively, from Megazyme. The GOS formed was calculated based on the mass balance:

3 $$\left[GOS\right]\left(mg/mL\right)={\left[Lac\right]}_{initial}-\left[Glc\right]-\left[Gal\right]-{\left[Lac\right]}_{unconverted}$$

with [Glc]: glucose concentration (mg/mL); [Gal]: galactose concentration (mg/mL); [Lac]: lactose concentration (mg/mL).

Comparison of the GOS formed by the wild-type enzyme (*Bbre*βgal-III) and the variants was performed by plotting the GOS yields against lactose conversion (%), which was calculated as:

4 $$lactose\;conversion\;\left(\%\right)=\frac{{\left[Lac\right]}_{initial}-{\left[Lac\right]}_{unconverted}}{{\left[Lac\right]}_{initial}}$$

with [Lac]: lactose concentration (mg/mL)

The individual GOS components were determined by HPAEC. High-performance size exclusion chromatography with UV-based detection (HPSEC-UV) was conducted for the size distribution analysis of the GOS mixtures.

### Substrate Specificity in Hydrolysis Mode

One mg/mL of each galactoside, including β-d-Gal*p*-(1→3)-d-Gal, β-d-Gal*p*-(1→4)-d-Gal, β-d-Gal*p*-(1→6)-d-Gal, β-d-Gal*p*-(1→3)-d-Glc, β-d-Gal*p*-(1→4)-d-Glc (lactose), β-d-Gal*p*-(1→6)-d-Glc (allolactose), β-d-Gal*p*-(1→3)-d-Lac, β-d-Gal*p*-(1→6)-d-Lac, and β-d-Galp-(1→4)-d-Lac, was incubated at 30 °C with 0.1 U of the wild-type or mutant enzymes per mL of the reaction mixture. Samples were taken after 30 min of reaction, and the reactions were stopped by incubation at 95 °C for 5 min. The remaining concentration of each galactoside after the reaction was quantified by HPAEC-PAD using authentic GOS purchased from BioSynth (Berkshire, UK) or Dextra UK (Reading, UK) as external standards. The rates of the hydrolysis of each galactoside by the wild-type or mutant enzymes are expressed relatively to the hydrolysis of lactose (as 100%) after 30 min under the same conditions.

### Effects of Temperature, pH, Enzyme Dosage, and Sugar Donor Concentration on Transferase Activity

To investigate the effects of various factors on the transgalactosylation reaction, both the global enzyme activities and transgalactosylation activities were measured under varying reaction conditions: temperatures (20–60 °C), pH (4–8; using 50 mM phosphate citrate buffer for pH 4–5 and 50 mM potassium phosphate buffer for pH 6–8), enzyme dosages (1–100 U/mL), and lactose concentrations (50–600 mM). Moreover, thermostability was evaluated by incubating the enzyme in 50 mM NaPB (pH 6.5) at temperatures ranging from 40 to 70 °C for 1 h. The residual activities were then measured. For transferase activity determination, the measurements were conducted under initial velocity conditions by incubating 20 μL of enzyme with 480 μL of substrate for 10 min. The reaction was stopped by heating at 95 °C for 10 min. The concentrations of d-glucose and d-galactose in the reaction mixture were measured by using the d-Glucose Assay Kit (GOPOD Format) and the l-Arabinose/d-Galactose Assay Kit from Megazyme. The transferase activity was calculated using the following equation:

$$transferase\;activity=\frac{\left[Glc\right]}{\left[Gal\right]}$$ 5

with [Glc]: glucose concentration (mg/mL); [Gal]: galactose concentration (mg/mL).

### Thin-Layer Chromatography

A diluted sample of sugars (2 μL, ∼10 g/L) was applied to high-performance TLC silica plates (Kieselgel 60 F245, Merck). The mobile phase was prepared as a mixture of *n*-butanol/*n*-propanol/ethanol/water (2/3/3/2). After separation of the sugars, the thymol reagent was used for spot detection.

### High-Performance Anion-Exchange Chromatography

Individual GOS components were identified and quantitated by HPAEC with pulsed amperometric detection (HPAEC-PAD) as previously described.^32,58^ Briefly, HPAEC-PAD analysis was carried out on a Dionex DX-500 system consisting of a GP50 gradient pump, an ED 40 electrochemical detector with a gold working electrode, and a Ag/AgCl reference electrode. Separations were performed at a rate of 1 mL/min on a CarboPac PA-1 column (4 mm × 250 mm) connected to a CarboPac PA-1 guard column (Dionex). Two different combinations of four eluents were used for the effective GOS separation. Eluent A (100 mM NaOH), eluent B (water), eluent C (100 mM NaOH and 500 mM NaOAc), and eluent D (100 mM NaOH and 50 mM NaOAc) were mixed to form the following gradients: gradient 1, 100% A from 0 to 20 min; 0 to 100% D from 20 to 35 min; 100% C from 35 to 45 min; gradient 2, 15% A and 85% B from 0 to 40 min; 100% C from 40 to 50 min. Authentic GOS and lactose including β-d-Gal*p*-(1→3)-d-Gal, β-d-Gal*p*-(1→6)-d-Gal, β-d-Gal*p*-(1→3)-d-Glc, β-d-Gal*p*-(1→4)-d-Glc (lactose), β-d-Gal*p*-(1→6)-d-Glc, β-d-Gal*p*-(1→3)-d-Lac, β-d-Gal*p*-(1→6)-d-Lac, and β-d-Galp-(1→4)-d-Lac purchased from BioSynth or Dextra UK were used as external standards.

### High-Performance Size Exclusion Chromatography with UV-Based Detection

Size distribution analysis of the GOS mixtures was performed on an XK 16/100 column (1.6 × 100 cm, Cyptiva, MA) packed with Bio-Gel P2 Gel (Bio-Rad, CA, USA). The column was eluted with distilled water at a flow rate of 0.15 mL/min.^3^ The elution was monitored by UV detection at 190 nm to detect the transgalactosylation products. Fractions containing different DP of GOS were then pooled and lyophilized for NMR analysis.

### NMR Spectroscopy

NMR spectra were recorded on a Bruker AVANCE III 600 equipped with a room temperature probehead or an AVANCE Neo 600 instrument with a Prodigy cryoprobehead (600.22 MHz for ^1^H, 150.93 MHz for ^13^C) using standard Bruker NMR software. ^1^H spectra were recorded in D_2_O at 300 K. Assignments were based on COSY, HSQC, HMBC, and TOCSY data. Spectra were referenced internally to acetone (^1^H 2.225 ppm, ^13^C 31.08 ppm).

### MALDI-TOF

MALDI-TOF spectra were recorded on a Bruker Autoflex Speed instrument. Samples were cospotted on an MTP-384 ground steel target plate with a 2,5-dihydroxybenzoic acid (DHB)-based matrix DHB (1.0 mg), 10% trifluoroacetic acid (aq, 1.0 μL), and acetonitrile/water: 1/1 (100 μL).

### Statistical Analysis

All measurements were carried out in at least duplicates, and the data are expressed as the mean ± SD (standard deviation). The standard deviations were always less than 5%.

## Supplementary Material



## Notes

Primers used in this study (Table S1); Superimposition of active-site amino acid residues of GH42 *Bbre*βgal-III onto GH42 *Bi*Bga42A from *B. longum* subsp. *infantis* ATCC 15697 and GH42 BbgII from *B. bifidum* S17 (Figure S1); Site-saturation mutagenesis of Arg121 in *Bbre*βgal-III from molecular cloning to protein expression and purification (Figure S2); Distribution of R121 variants in terms of relative enzyme and transgalactosylation activities to wild-type *Bbre*βgal-III (Figure S3); Representative HPAEC-PAD chromatograms for GOS analysis (Figure S4); Size distribution of GOS formed during the lactose conversion by *Bbre*βgal-III-R121C using HPSEC-UV (Figure S5); (A) ^1^H NMR spectrum of the disaccharide fraction; (B) Comparison of ^1^H NMR spectra of the trisaccharide fraction of *Bbre*βgal-III-R121C and *Bbre*βgal-III-wild type (Figure S6); ^1^H NMR comparison of penta- (A) and tetrasaccharide (B) fractions (Figure S7); Occurrence of hydrogen bonds during the molecular dynamics simulations (Figure S8); Representation of the number of waters associated with the tunnels as a function of simulation time (Figure S9); Comparison of the water tunnels of two GH42 β-galactosidases from *B. bifidum* S17 and *B. longum* subsp. *infantis* ATCC 15697 with *Bbre*βgal-III (Figure S10); Amino acids lining the water tunnel A of GH42 β-galactosidases found in the UniProt database (Figure S11); and pH dependency of enzyme activity (Figure S12) (PDF)
