# Supplementary material for: Unveiling the Transgalactosylation Switch of a GH42 β‑Galactosidase from the Infant Isolate Bifidobacterium breve DSM20213
Source: ACS Catal. 2026 Jan 23;16(3):2669–86. doi: 10.1021/acscatal.5c08164 (PMC12887932; doi:10.1021/acscatal.5c08164)
Supplement: Supplementary file 1 [file cs5c08164_si_001.pdf]

Unveiling the transgalactosylation switch of a GH42 β-galactosidase from the infant isolate *Bifidobacterium breve* DSM20213

Konlarat Phirom-on[a,b], Khanh-Trang Vu-Le[a,c], Leander Sützl[a], Benedikt Lehner[a], David Whelan[a], Lucile Guerent[a], Irene Pasini[d], Marc Schuh[b,e], Anita de Ruiter[e], Markus Blaukopf[d], Dietmar Haltrich[a,b], Chris Oostenbrink[b,e], Thu-Ha Nguyen[a,*]

[a] Food Biotechnology Laboratory, Institute of Food Technology, Department of Biotechnology and Food Science, BOKU University, Muthgasse 18, A-1190 Vienna, Austria

[b] Doctoral Programme BioToP - Biomolecular Technology of Proteins, BOKU University, Muthgasse 18, A-1190 Vienna, Austria

[c] Faculty of Biology and Environmental Science, The University of Danang - University of Science and Education, Danang, Vietnam

[d] Institute of Organic Chemistry, Department of Natural Sciences and Sustainable Resources, BOKU University, Muthgasse 18, A-1190 Vienna, Austria

[e] Institute for Molecular Modeling and Simulation, Department of Natural Sciences and Sustainable Resources, BOKU University, Muthgasse 18, A-1190 Vienna, Austria

[*] Corresponding author

E-mail address: thu-ha.nguyen@boku.ac.at

Table S1 Primers used in this study

(A) Site-saturated mutagenesis at position Arg121

Primer	Sequence (5′→3′)			Amino acid *in italics*
Ala_Fw	CCAGCCCGGTGCC	*GCG*	CAGCACTG	Alanine
Asn_Fw	CCAGCCCGGTGCC	*AAC*	CAGCACTG	Asparagine
Asp_Fw	CCAGCCCGGTGCC	*GAT*	CAGCACTG	Aspartic acid
Cys_Fw	CCAGCCCGGTGCC	*TGC*	CAGCACTG	Cysteine
Glu_Fw	CCAGCCCGGTGCC	*GAA*	CAGCACTG	Glutamic acid
Gln_Fw	CCAGCCCGGTGCC	*CAG*	CAGCACTG	Glutamine
Gly_Fw	CCAGCCCGGTGCC	*GGC*	CAGCACTG	Glycine
His_Fw	CCAGCCCGGTGCC	*CAT*	CAGCACTG	Histidine
Ile_Fw	CCAGCCCGGTGCC	*ATT*	CAGCACTG	Isoleucine
Leu_Fw	CCAGCCCGGTGCC	*CTG*	CAGCACTG	Leucine
Lys_Fw	CCAGCCCGGTGCC	*AAA*	CAGCACTG	Lysine
Met_Fw	CCAGCCCGGTGCC	*ATG*	CAGCACTG	Methionine
Phe_Fw	CCAGCCCGGTGCC	*TTT*	CAGCACTG	Phenylalanine
Pro_Fw	CCAGCCCGGTGCC	*CCG*	CAGCACTG	Proline
Ser_Fw	CCAGCCCGGTGCC	*AGC*	CAGCACTG	Serine
Thr_Fw	CCAGCCCGGTGCC	*ACC*	CAGCACTG	Threonine
Trp_Fw	CCAGCCCGGTGCC	*TGG*	CAGCACTG	Tryptophan
Tyr_Fw	CCAGCCCGGTGCC	*TAT*	CAGCACTG	Tyrosine
Val_Fw	CCAGCCCGGTGCC	*GTG*	CAGCACTG	Valine
All_Rv	GGCACCGGGCTGGCAGACGTCG			

Table S1 Primers used in this study (cont'd)

(B) Water tunnel

**Primer**	**Sequence (5′→3′)**
F199A_Fw	GGGCACCGCC *GCG* TGGGCGC
F199_Rv	GGCGGTGCCCCAGGCGTCATTG
F356A_Fw	GCCATCTGCTAC *GCG* CAGTGGCGC
F356_Rv	GTAGCAGATGGCGTCGGAGCCC
F55A_Fw	TCCGTAGCCATC *GCG* TCTTGGGCC
F55A_Rv	GATGGCTACGGAGACGAGGTTGACG
Q29A_Fw	ATTACAACCCCGAC *GCG* TGGCCTGAG
Q29A_Rv	GTCGGGGTTGTAATCGCCGCC
S56A_Fw	GTAGCCATCTTC *GCG* TGGGCCAAGC
S56A_Rv	GAAGATGGCTACGGAGACGAGGTTG
W200A_Fw	GGGCACCGCCTTC *GCG* GCGCAG
W200_Rv	GAAGGCGGTGCCCCAGGCGTCATTGAC

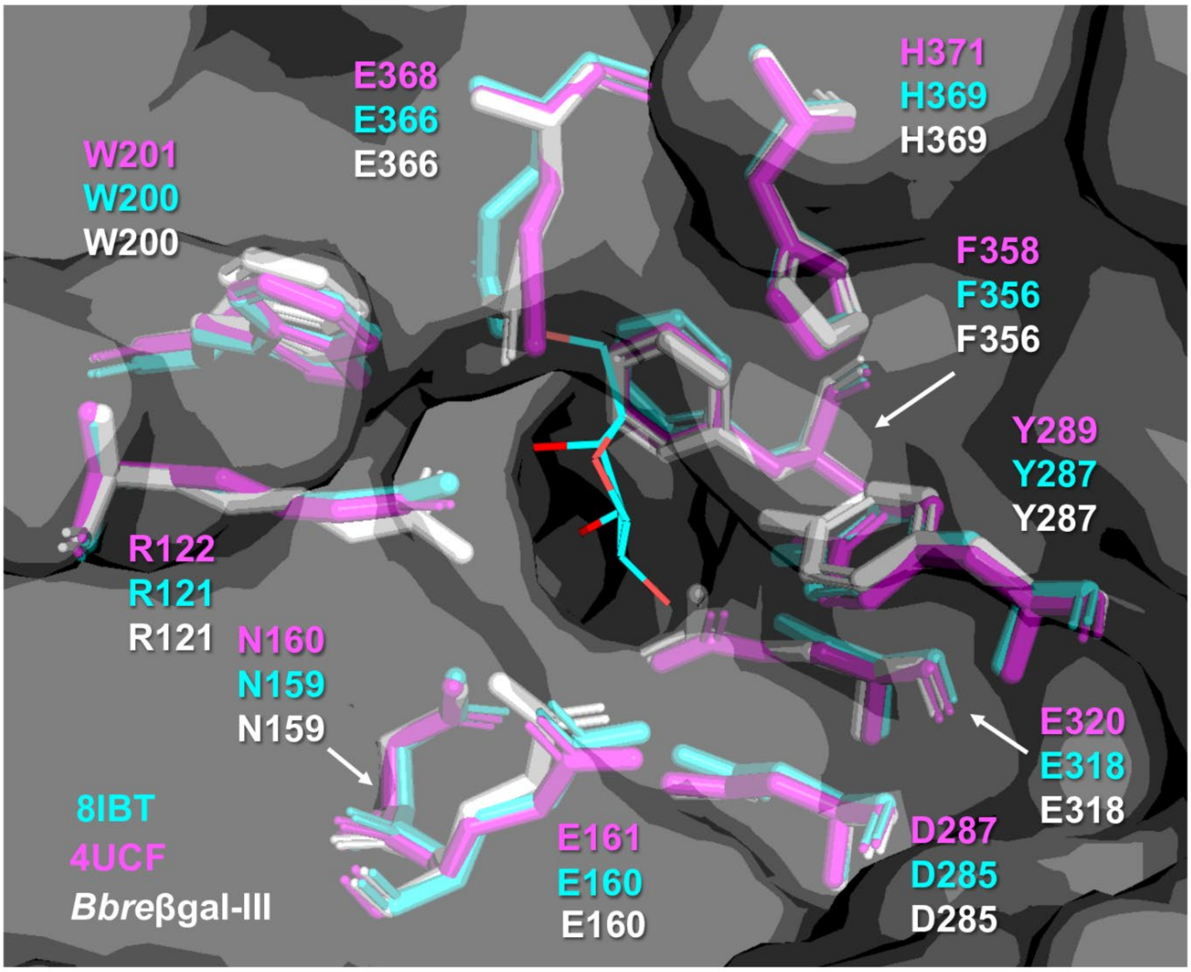

Figure S1. Superimposition of active site amino acid residues of GH42 *Bbre*βgal-III (white) onto GH42 *Bi*Bga42A from *Bifidobacterium longum* subspecies *infantis* ATCC 15697 (PDB: 8IBT, blue) and GH42 BbgII from *Bifidobacterium bifidum* S17 (PDB: 4UCF, purple)

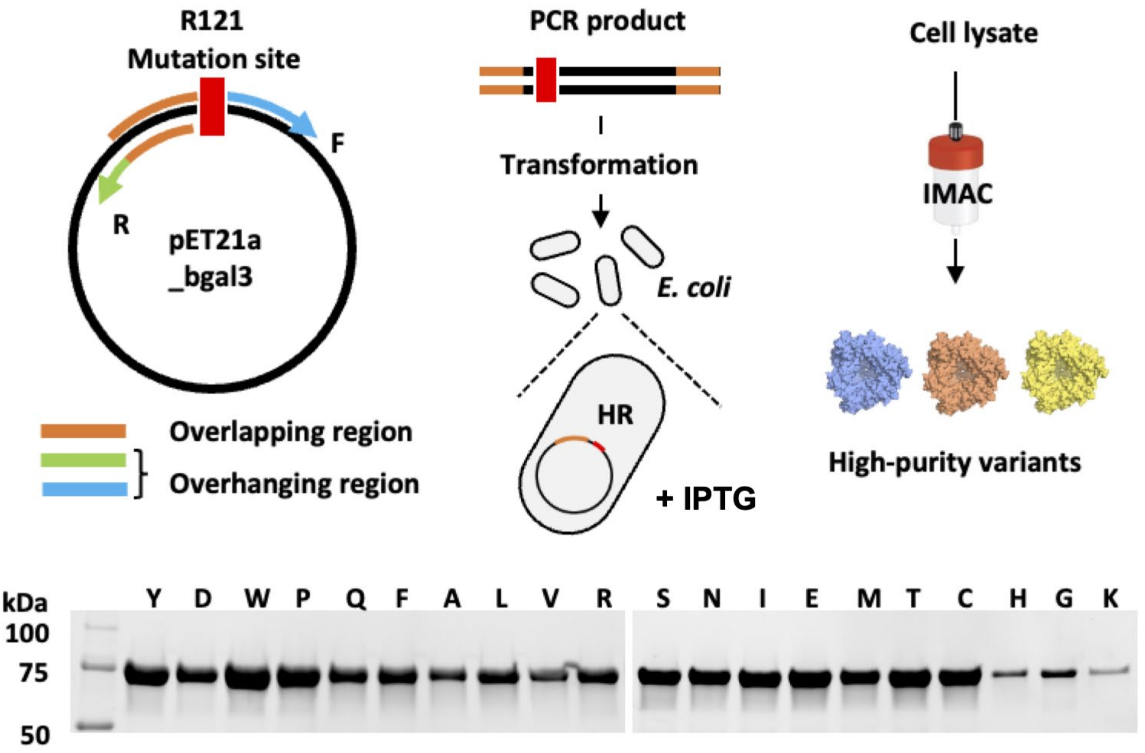

Figure S2. Site-saturation mutagenesis of Arg121 in *Bbre*βgal-III from molecular cloning to protein expression and purification. The recombinant plasmid pET21a-Bbreβgal-III containing the gene that encodes *Bbre*βgal-III was constructed from our previous work[27]. The whole pET21a-Bbreβgal-III plasmid was used as a template for PCR-based site-saturation mutagenesis. The PCR products were then transformed into *E. coli* BL21 for protein expression induced with isopropyl-β-D-thiogalactopyranoside (IPTG). The expressed cells were harvested and lysed to obtain crude enzymes. The mutants of *Bbre*βgal-III were purified using an immobilized metal affinity chromatography (IMAC) column, and their purity was assessed by SDS-PAGE.

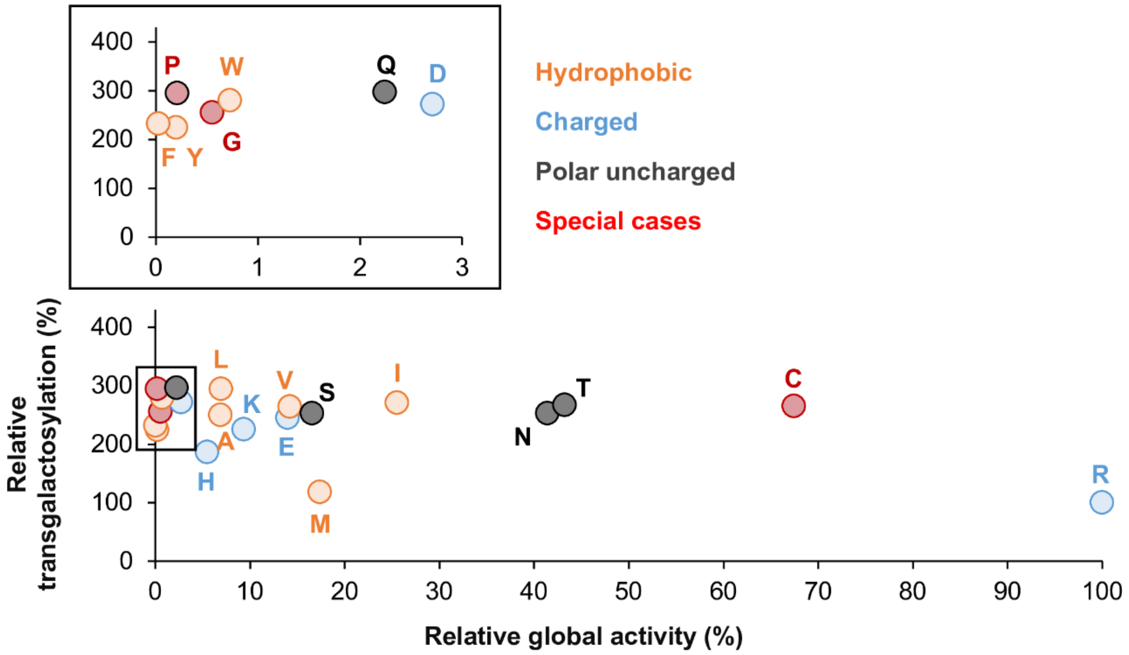

Figure S3. Distribution of R121 variants in terms of relative enzyme and transgalactosylation activities to the wild-type *Bbre*βgal-III.

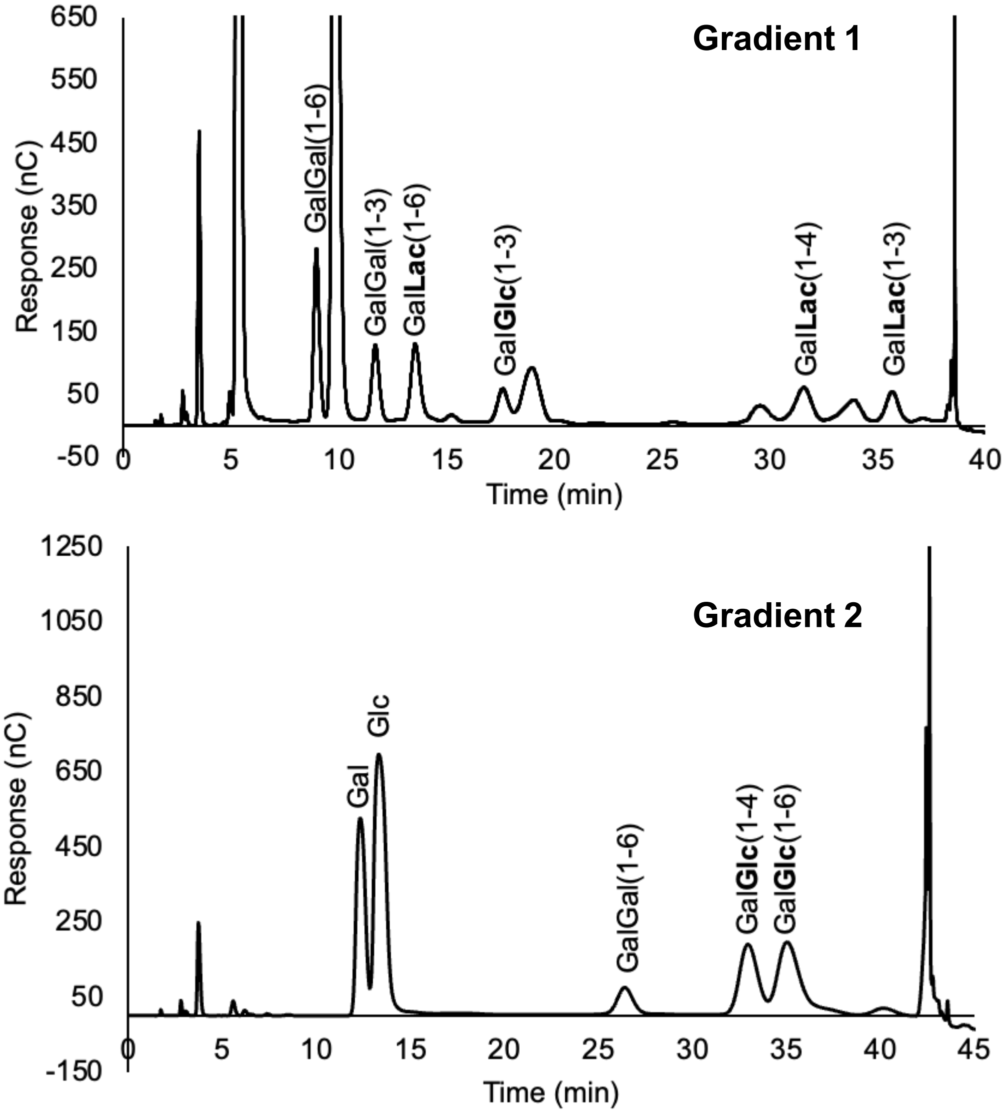

Figure S4. Representative HPAEC-PAD chromatograms for GOS analysis. Two different combinations of four eluents were used for effective GOS separation. Eluent A (100 mM NaOH), eluent B (water), eluent C (100 mM NaOH and 500 mM NaOAc), and eluent D (100 mM NaOH and 50 mM NaOAc) were mixed to form the following gradients: gradient 1, 100% A from 0 to 20 min; 0 to 100% D from 20 to 35 min; 100% C from 35 to 45 min, and gradient 2, 15% A and 85% B from 0 to 40 min; 100% C from 40 to 50 min. Authentic oligosaccharides including β-D-Gal*p*-(1→3)-D-Gal, β-D-Gal*p*-(1→6)-D-Gal, β-D-Gal*p*-(1→3)-D-Glc, β-D-Gal*p*-(1→4)-D-Glc, β-D-Gal*p*-(1→6)-D-Glc, β-D-Gal*p*-(1→3)-D-Lac, β-D-Gal*p*-(1→6)-D-Lac, and β-D-Galp-(1→4)-D-Lac were used as external standards.

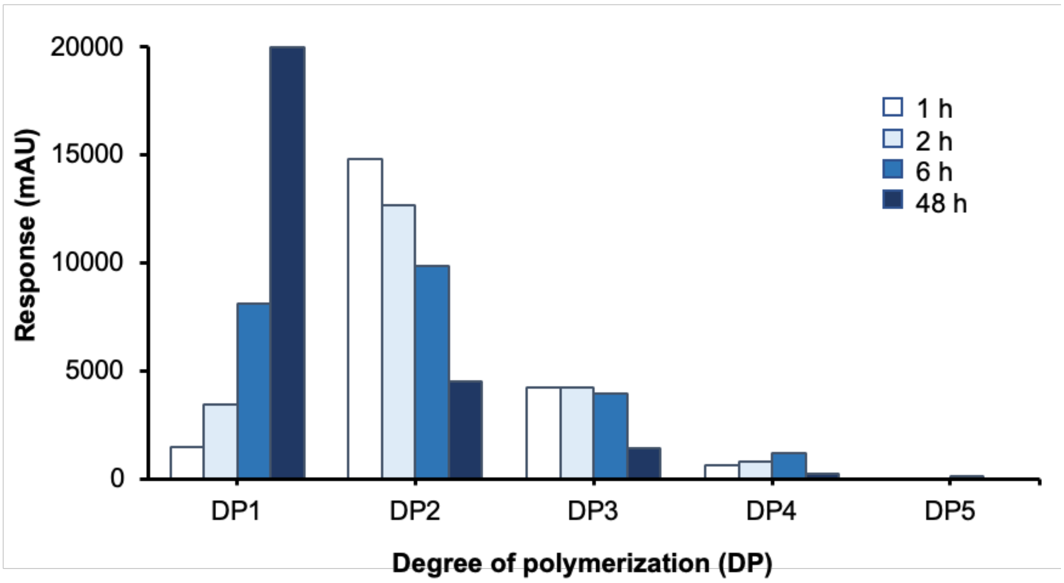

Figure S5. Size distribution of GOS formed during the lactose conversion by *Bbre*βgal-III-R121C using HPSEC-UV (high performance size exclusion chromatography with UV-based detection). DP: degree of polymerization

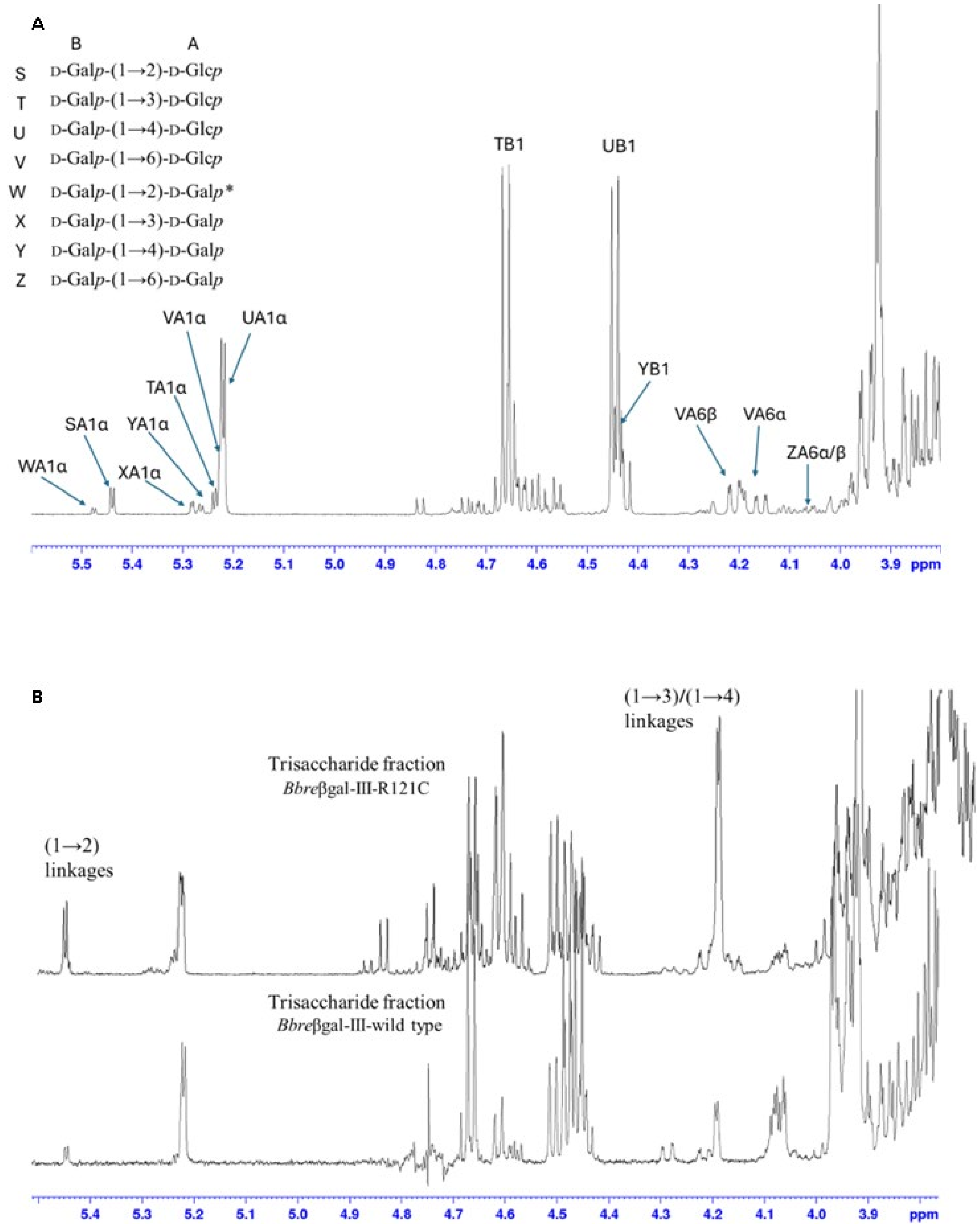

Figure S6. (A) ^{1}H NMR spectrum of the disaccharide fraction. Reporter signals are indicated by arrows. **(B)** Comparison of ^{1}H NMR spectra of the trisaccharide fraction of *Bbre*βgal-III-R121C and *Bbre*βgal-III-wild type. Signals indicative for 1-2 and 1-3/4 linkages are highlighted. Both spectra were recorded in D_2O, HOD signal was removed with a 1D DOSY pulse sequence (ledbpgp2s1d, 50% gradient strength)

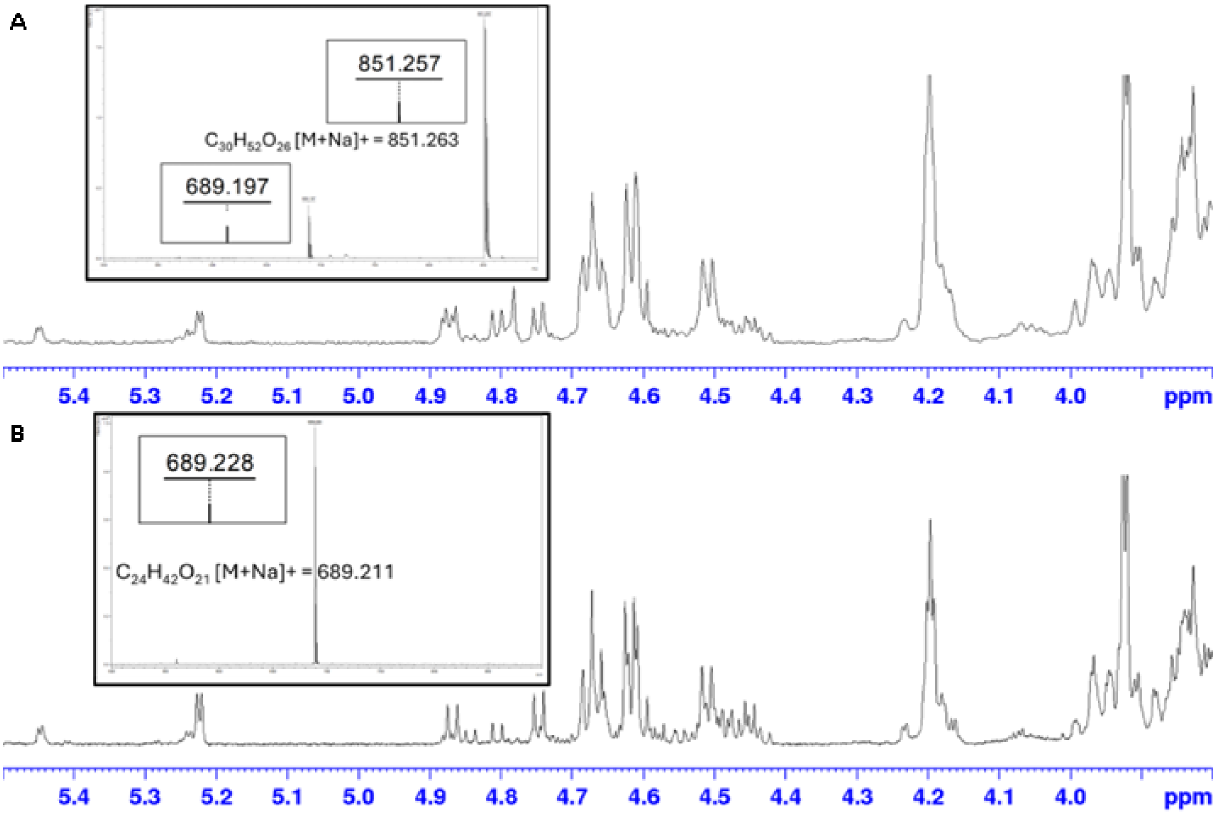

Figure S7. ^{1}H NMR comparison of penta- (**A**) and tetrasaccharide (**B**) fractions. MALDI spectra for both fractions are displayed in framed boxes. Both spectra were recorded in D_2O, HOD signal was removed with a 1D DOSY pulse sequence (ledbpgp2s1d, 50% gradient strength).

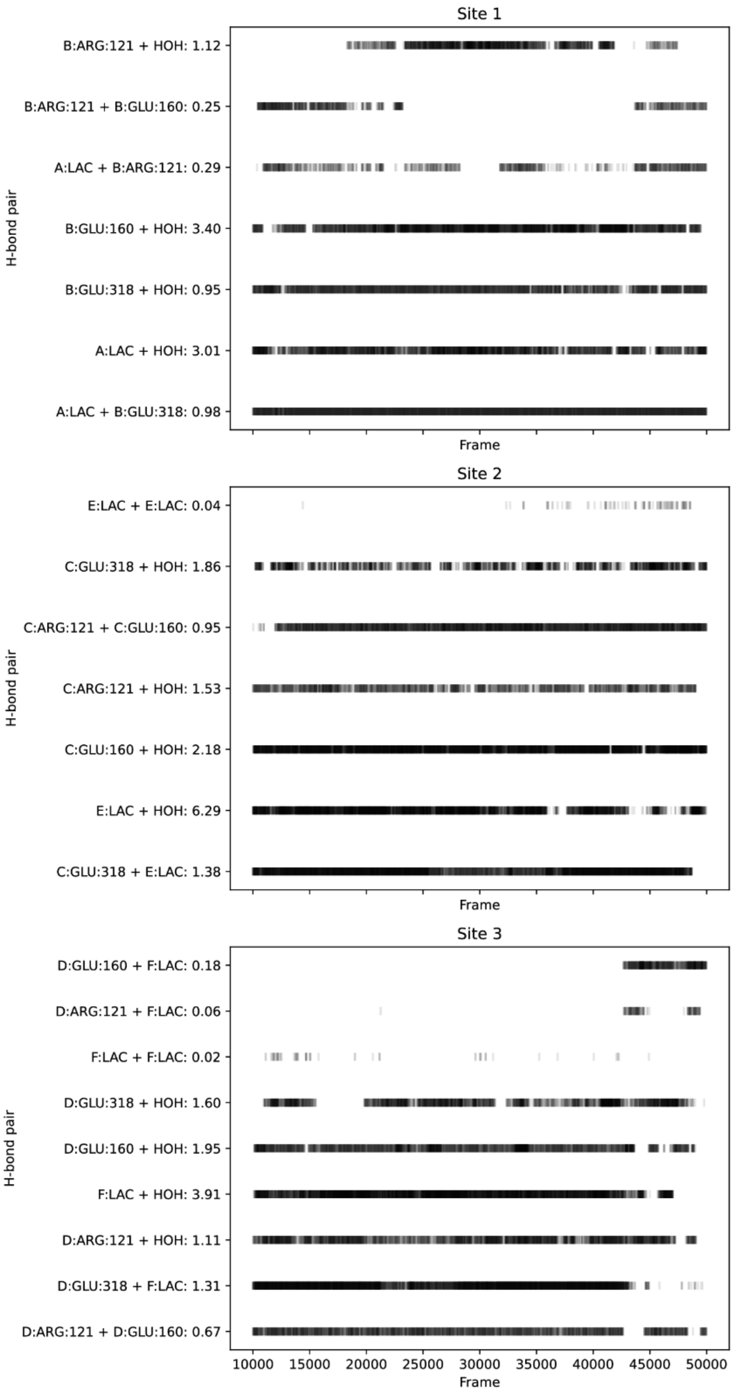

Figure S8. Occurrence of hydrogen bonds during the molecular dynamics simulations. A hydrogen bond analysis was performed for frames from the simulation, sampled every 10 ps, leading to 50000 frames. The labels on the *x*-axis indicate the specific hydrogen bonding pair, together with the average number of hydrogen bonds observed. In case multiple hydrogen bonds are possible for one pair, the occurrence of these hydrogen bonds was joined in the analysis.

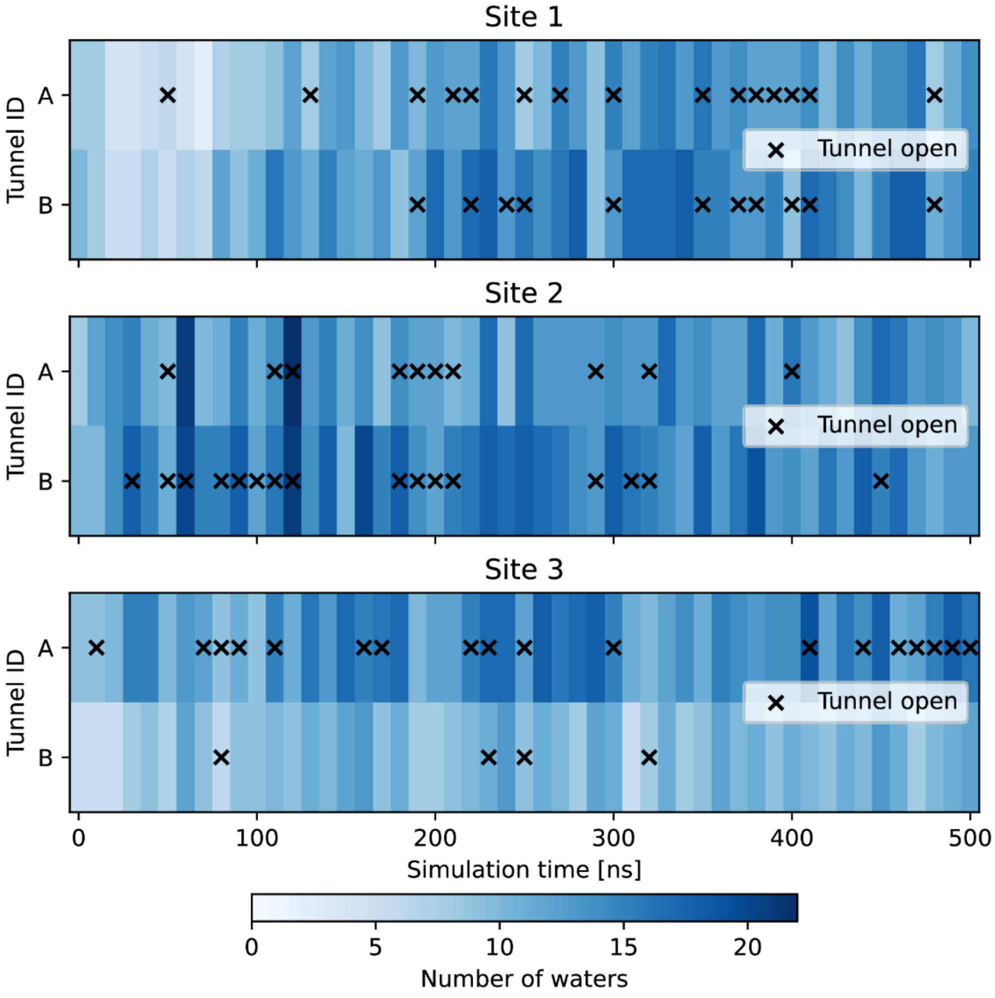

Figure S9. Representation of the number of waters associated to the tunnels (indicated by color) as a function of simulation time. A black cross indicates that the tunnel was detected and open in that timepoint in the simulation as per the Caver analysis. Tunnel IDs correspond to those defined in Figure 8.

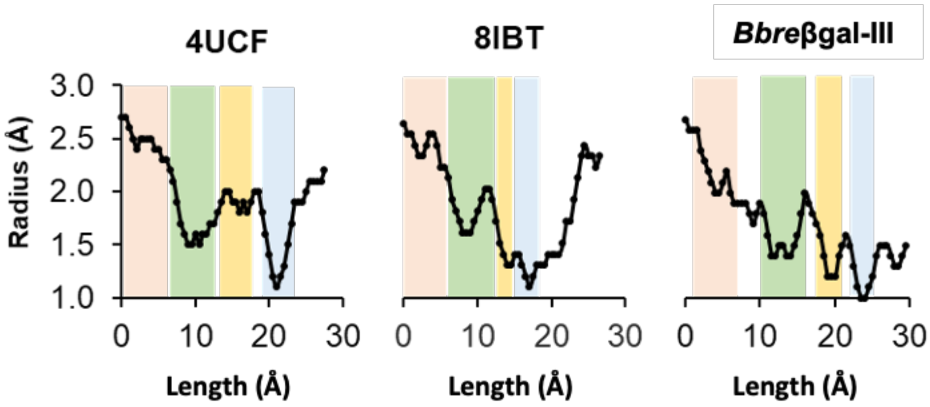

Figure S10. Comparison of the water tunnels of two GH42 β-galactosidases from *B. bifidum* S17 (PDB: 4UCF) and *B. longum* subspecies *infantis* ATCC 15697 (PDB ID: 8IBT) with *Bbre*βgal-III. Three potential gatekeeper residues are identified in both proteins.

A

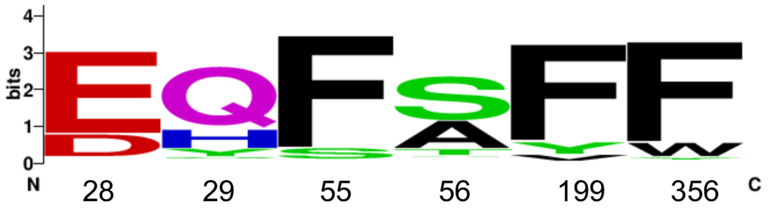

B

Position	Amino acid (Top 3)
28	E=73.4%, D=20.5%, K=1.4%
29	Q=63.9%, H=20.2%, Y=9.0%
55	F=87.1%, S=9.4%, L=1.1%
56	S=51.1%, A=32.4%, T=7.9%
199	F=80.9%, Y=10.9%, V=6.0%
356	F=82.2%, W=12.0%, Y=2.9%

Figure S11. The amino acids lining the water tunnel A of GH42 β-galactosidases found in the UniProt database. (**A**) Sequence logo analysis; (**B**) Top 3 amino acid substitutions at the positions lining the water tunnel A

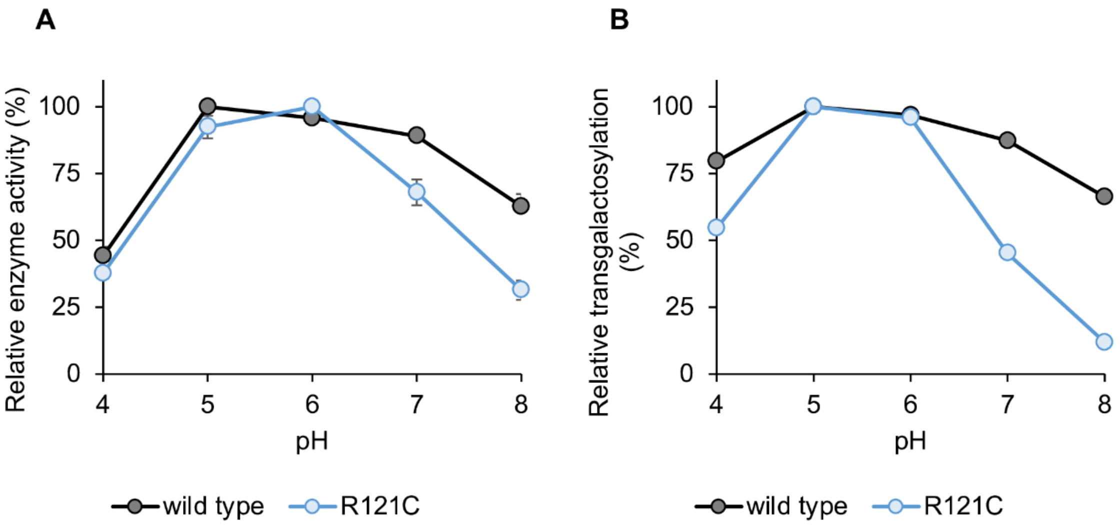

Figure S12. pH dependency of enzyme activity. Relative enzyme activity (**A**) and relative transgalactosylation (**B**) of wild-type *Bbre*βgal-III and *Bbre*βgal-III-R121C.
